# Skin and gut microbiota composition and immune regulatory response differentiate IgE and non-IgE cow’s milk allergy patients with atopic dermatitis

**DOI:** 10.1016/j.isci.2025.113943

**Published:** 2025-11-04

**Authors:** Tomas Thon, Eliska Kopelentova, Dagmar Srutkova, Stepan Coufal, Jakub Kreisinger, Filip Rob, Zuzana Reiss, Miloslav Kverka, Stepanka Capkova, Jana Cadova, Lucie Bulantova, Vojtech Bystry, Anna Sediva, Helena Tlaskalova-Hogenova, Zuzana Jiraskova Zakostelska, Andrea Polouckova

**Affiliations:** 1 Laboratory of Cellular and Molecular Immunology, Institute of Microbiology of the Czech Academy of Sciences, Prague, Czech Republic; 2 Department of Immunology, 2nd Faculty of Medicine, Charles University and Motol University Hospital, Prague, Czech Republic; 3 Laboratory of Gnotobiology, Institute of Microbiology of the Czech Academy of Sciences, Novy Hradek, Czech Republic; 4 Laboratory of Animal Evolutionary Biology, Faculty of Science, Department of Zoology, Charles University, Prague, Czech Republic; 5 Department of Dermatovenerology, Second Faculty of Medicine, Charles University, Bulovka University Hospital, Prague, Czech Republic; 6 Department of Paediatric Dermatology, Motol University Hospital, Prague, Czech Republic; 7 CEITEC-Central European Institute of Technology, Masaryk University, Brno, Czech Republic; 8 Institute of Clinical Immunology and Allergology, First Faculty of Medicine, Charles University and General University Hospital in Prague, Prague, Czech Republic

**Keywords:** Immunology, Microbiology

## Abstract

Precise identification of food allergy and atopic dermatitis (AD) endotypes in infants is needed to target treatments effectively. Therefore, we investigated markers associated with changes in the microbiota and immune responses within the gut-skin axis of immunoglobulin E (IgE) and non-IgE-mediated cow’s milk allergy (CMA) patients with AD. We report that the skin microbiota of patients with IgE CMA differs significantly from healthy controls (HCs) and from patients with non-IgE CMA, despite similar AD severity. Regarding the immune response to bacteria, we found a significant increase in soluble CD14 in patients with non-IgE CMA compared to patients with IgE CMA. Patients with a non-IgE CMA have more regulatory T cells in their blood that migrate into the intestine than patients with IgE CMA. These findings provide insights into the complex interplay between the damaged epithelial barrier, microbiome, and immune responses in CMA patients with AD.

## Highlights

• Skin microbiota differs between atopic dermatitis patients with IgE vs. non-IgE cow’s milk allergy
• Non-IgE cow’s milk allergy patients have higher serum soluble CD14 than IgE patients
• IgE cow’s milk allergy patients have higher serum soluble E-FABP than non-IgE patients
• Non-IgE cow’s milk allergy patients have more Tregs migrating to gut than IgE patients

## Graphical abstract

**Figure:**
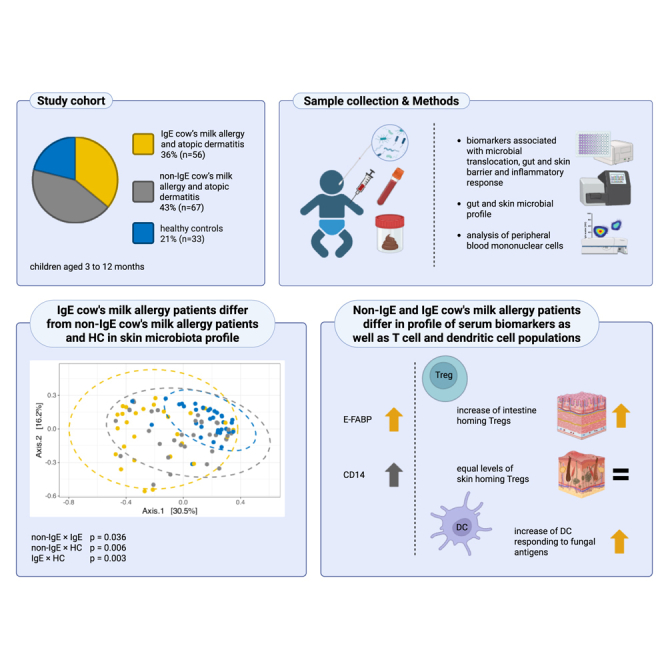

## Introduction

Food allergy (FA) in children is a serious health problem with increasing prevalence affecting around 10% of children worldwide.^1^ FA is defined as immune-mediated hypersensitivity reaction to a specific food antigen, with cow’s milk allergy (CMA) being among the earliest and most prevalent due to its early introduction into the diet.^2^^,^^3^ Depending on the presence or absence of specific immunoglobulin E (IgE) antibodies, FA may be divided into two endotypes either IgE-mediated or non-IgE-mediated.^2^ There are general differences in allergic symptoms between IgE and non-IgE FA. The IgE allergic reactions are immediate, and caused by food-specific IgE, which can usually be detected by clinical tests to confirm the diagnosis of FA.^4^ These food-specific IgEs bind to high-affinity receptors for the Fc region of IgE on mast cells, leading to the release of histamine and other mediators that cause typical symptoms such as vasodilation, hives, angioedema, low blood pressure, and smooth muscle constriction, resulting in diarrhea or vomiting.^5^ In contrast to IgE FA endotype, which can lead to systemic immune response and even anaphylaxis of the organism, non-IgE FA primarily affects the gastrointestinal tract. The symptoms of non-IgE FA are usually delayed as compared to IgE FA. Therefore, it is more difficult to determine the association between offending food and the symptoms, which are very similar to other common diseases, especially in the first year of life, such as colic, gastroesophageal reflux, diarrhea, and eczema. The urgent lack of easily accessible blood or skin marker testing also contributes to the problem.^6^ Both types of allergies are associated with the occurrence of atopic dermatitis (AD), suggesting that AD plays an important role in the early activation of the pro-allergic immune response.^7^^,^^8^ In addition, several endotypes of AD have recently been described. They are defined by distinct biological drivers (e.g., amount and specificity of IgE, type of T helper (Th) cell response, changes in filaggrin presence and function, amounts of antimicrobial protein, and diversity of microbial colonization) rather than by clinical appearance.^9^^,^^10^^,^^11^ Current dual-allergen exposure hypothesis assumes that skin barrier damage, which is often the case in AD patients, could allow the penetration of food antigens through the skin. In the absence of prior oral tolerance to certain foods, skin exposure leads to food-antigen sensitization characterized by a deviation of T cells toward a pro-allergic Th2 type and subsequent allergy development in predisposed individuals.^12^^,^^13^ In contrast, early oral exposure to food antigens promotes immune tolerance, primarily through the induction of regulatory T cell subsets.^14^ Moreover, growing evidence supports the existence of a skin-gut axis, whereby skin inflammation can influence the intestinal epithelial and immune landscape, potentially driving Th2 response and contributing to the development of FA.^15^ A damaged and leaky epithelial barrier of the skin and gut can alter the microbiome through the colonization of opportunistic pathobionts, local inflammation, and decreased diversity in microbiota leading to microbial dysbiosis.^16^ A strong correlation has been described between severe AD and a lower bacterial diversity of the skin.^17^ In addition, the skin lesions of more than 90% of patients with AD are colonized with pathogenic *Staphylococcus aureus*, whereas this bacterium is rarely found on the skin of healthy individuals.^18^ Delta-toxin from *S. aureus* was identified as a mast cell degranulation-inducing factor that could contribute to IgE-mediated allergic diseases such as AD or FA.^19^^,^^20^^,^^21^ Further, Azad et al. described that low gut microbiota richness and increased Enterobacteriaceae/Bacteroidaceae ratio in early infancy are associated with later food sensitization, suggesting that early gut colonization contributes to the development of atopic diseases, including AD and FA.^22^ Sjodin et al. showed an underrepresentation of *Ruminococcus*, *Bacteroides*, *Prevotella*, and *Coprococcus* species in IgE-associated allergic children compared to non-allergic children from infancy to school age.^23^ On the other hand, patients with AD harbor less diverse gut microbiota, with a lower abundance of *Bifidobacterium* spp. while being more frequently colonized with *S. aureus*, certain clostridia, and *Escherichia coli*.^24^ These changes in gut microbiota composition could lead to alterations in intestinal barrier function predisposing to local and systemic inflammatory responses.^25^^,^^26^^,^^27^ Imbalances in commensal gut microbiota in early life may lead to oral tolerance impairment, which predispose toward IgE or non-IgE FA and AD.^28^

There is enormous interest in identifying the biomarkers that lead to the different clinical endotypes of CMA and AD, in the hope that this will lead to personalized treatments with better outcomes than are possible with current non-specific approaches. Given that skin and gut barrier dysfunction contributes to the development of both AD and FA,^29^ we investigated serum markers previously found to be associated with altered barrier and immune response in inflammatory bowel disease (IBD) patients.^30^^,^^31^ Our results showed that these immune molecules contribute to better characterization of CMA endotypes by distinguishing IgE and non-IgE CMA and AD with regard to microbiome composition, immune response, and epithelial barrier function. Moreover, we proved our hypothesis that skin and gut microbiota also significantly contribute to immune mechanisms underlying the IgE and non-IgE CMA pathogenesis in patients with AD. Taken together, we found markers associated with microbiota alterations in the gut-skin axis of AD patients with IgE or non-IgE CMA that could characterize disease endotypes.

## Results

### Gut microbiota of patients with non-IgE CMA differs from patients with IgE CMA and HC

**Figure 1:**
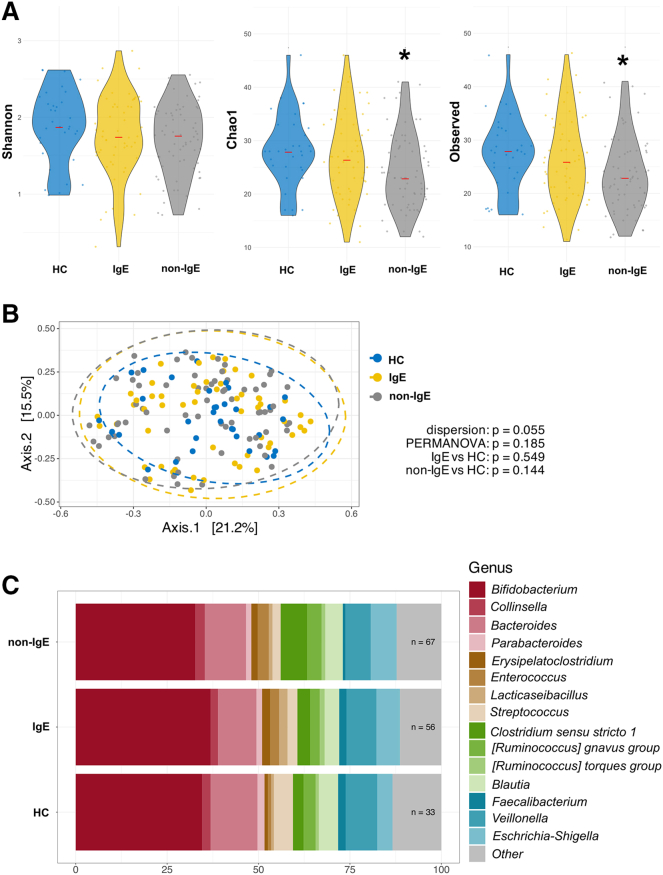
Gut bacteria of patients with non-IgE CMA differ from patients with IgE CMA and HC (A) Alpha diversity expressed as Shannon diversity index, Chao1, observed taxa. The significant differences among groups were tested using the Kruskal-Wallis test followed by Dunn’s test with Bonferroni correction. (B) Beta diversity was calculated using Bray-Curtis dissimilarities and visualized by principal coordinate analysis (PCoA) where each point represents one patient or healthy control and tested with permutational multivariate analysis of variance (PERMANOVA). (C) Abundances of the 15 most abundant bacterial taxa. These data display results without covariates for age and sex. Statistically significant differences between groups are marked with ∗ (∗*p* < 0.05).

**Figure 2:**
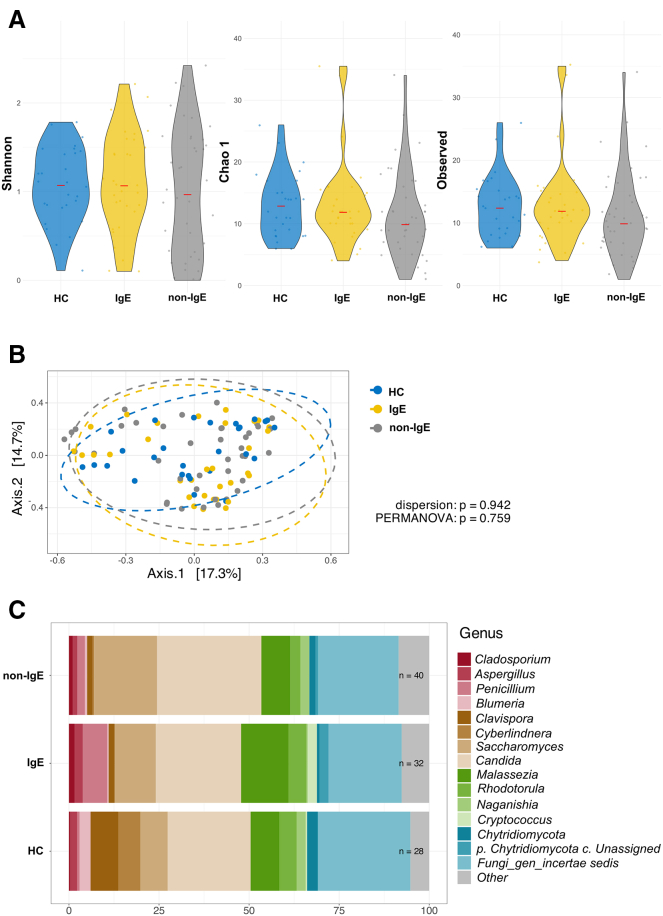
Gut fungi of patients with non-IgE CMA do not differ from patients with IgE CMA and HC (A) Alpha diversity expressed as Shannon diversity index, Chao1, observed taxa. The significant differences among groups were tested using the Kruskal-Wallis test followed by Dunn’s test with Bonferroni correction. (B) Beta diversity was calculated using Bray-Curtis dissimilarities and visualized by PCoA where each point represents one patient or healthy control and tested with PERMANOVA. (C) Abundances of the 15 most abundant fungal genera. These data display results without covariates for age and sex.

Here, we describe differences in the composition of the microbiota that distinguish these two immune types of allergies to cow’s milk compared to healthy control (HC). Our study cohort consists of 67 patients with non-IgE CMA and AD, 56 patients with IgE CMA and AD, and 33 HCs. The demographic and clinical characteristics are summarized in Table 1. We found no differences in the Shannon diversity index, which refers to the abundance and evenness of species present, in the bacterial composition of the gut between patients with IgE and non-IgE CMA. We found that patients with non-IgE CMA had lower gut alpha diversity when using the Chao1 index (species richness) and the number of observed taxa compared to HC. This pattern was not observed in patients with IgE CMA (Figure 1A). We found no significant differences in the beta diversity of the bacterial composition in the gut (Figure 1B). Relative proportional taxonomic differences in bacterial abundances between the IgE CMA, non-IgE CMA patients, and HC are shown in Figure 1C. Using analysis of compositions of microbiomes with bias correction (ANCOM-BC) with covariates for age and sex, we found a significantly lower abundance of genus *Streptococcus* in the gut of non-IgE CMA patients compared to HC (Table 2). Interestingly, there was a trend in lower abundance of family Streptococcaceae in the gut of non-IgE CMA patients compared to HC. We detected a non-significant trend toward higher abundance of families Enterococcaceae, Erysipelatoclostridiaceae, and Lactobacillaceae in the gut of IgE CMA patients compared to HC, which was not observed when comparing non-IgE CMA patients with HC (Table 2). Next, we found no statistically significant differences in alpha or beta diversity in fungal composition in the gut (Figures 2A and 2B), where significant differences were found only in abundances of several taxa (Table 3). Compared to HC, we found a lower abundance of the family Cryptococcaceae and Saccharomycetales_fam_Incentris, as well as genera *Cryptococcus* and *Candida* in patients with non-IgE CMA in the gut (Table 3).

**Table 1:** Summary of anthropometric and clinical parameters of patients and healthy controls in whom we analyzed the composition of the microbiota

	Patients with IgE cow’s milk allergy (*n* = 56)	Patients with non-IgE cow’s milk allergy (*n* = 67)	Healthy controls (*n* = 33)
Median of age in months (1.q; 3.q)	6.4 (5.0; 8.8)	5.5 (4.3; 6.9)	7.7 (6.2; 10.4)
Sex (%)			
Female	27 (48.2)	21 (31.3)	17 (51.5)
Male	29 (51.8)	46 (68.7)	16 (48.5)
SCORAD (%)			
Mild	18 (32.1)	31 (46.3)	–
Moderate	30 (53.6)	30 (44.8)	–
Severe	8 (14.3)	6 (9.0)	–
Other type of FA (%)			
Yes	53 (94.6)	40 (59.7)	–
No	3 (5.4)	27 (40.3)	–
Type of birth (%)			
Vaginal	42 (75.0)	50 (74.6)	25 (75.8)
Caesarean	14 (25.0)	17 (25.4)	5 (15.2)
NA	0 (0.0)	0 (0.0)	3 (9.1)
Breastfeeding (%)			
Yes	54 (96.4)	65 (97.0)	26 (78.8)
No	2 (3.6)	2 (3.0)	2 (6.1)
NA	0 (0.0)	0 (0.0)	5 (15.2)
Mothers’ history of allergic diseases (%)			
Yes	25 (44.6)	34 (50.7)	9 (27.3)
No	26 (46.4)	23 (34.3)	20 (60.6)
NA	5 (8.9)	10 (14.9)	4 (12.1)
Fathers’ history of allergic diseases (%)			
Yes	29 (51.8)	34 (50.7)	11 (33.3)
No	22 (39.3)	23 (34.3)	18 (54.5)
NA	5 (8.9)	10 (14.9)	4 (12.1)
Form of AD (%)			
Extrinsic	54 (96.4)	47 (70.1)	–
Intrinsic	2 (3.6)	20 (29.9)	–
Onset of tolerance to milk in 1 year (%)			
Yes	3 (5.4)	7 (10.4)	–
No	48 (85.7)	60 (89.6)	–
NA	5 (8.9)	0 (0)	–
Gastrointestinal manifestations (%)			
Yes	18 (32.1)	21 (31.3)	–
No	38 (67.9)	46 (68.7)	–
Stridor (%)			
Yes	13 (23.2)	9 (13.4)	–
No	41 (73.2)	58 (86.6)	–
NA	2 (3.6)	0 (0)	–
Allergic rhinitis (%)			
Yes	18 (32.1)	13 (19.4)	–
No	36 (64.3)	54 (80.6)	–
NA	2 (3.6)	0 (0)	–
Inhalant allergens (%)			
Yes	31 (55.4)	28 (41.8)	–
No	23 (41.1)	38 (56.7)	–
NA	2 (3.6)	1 (1.5)	–
Hypogammaglobulinemia (%)			
Yes	43 (76.8)	54 (80.6)	–
No	13 (23.2)	13 (19.4)	–
SCIg therapy (%)			
Yes	13 (23.2)	8 (11.9)	–
No	43 (76.8)	59 (88.1)	–

**Table 2:** Composition of the gut bacteria is altered in infants with IgE and non-IgE CMA compared to HC

Gut – bacteria				
Family	IgE vs. HC (adjusted *p* values)		non-IgE vs. HC (adjusted *p* values)	
Enterococcaceae	0.0852	↑	0.6805	
Erysipelatoclostridiaceae	0.0769	↑	0.8856	
Lactobacillaceae	0.0852	↑	0.9467	
Streptococcaceae	0.4766		0.0677	↓

Genus	IgE vs. HC (adjusted *p* values)		non-IgE vs. HC (adjusted *p* values)	

*Streptococcus*	0.4710		0.0423	↓

↑↓ indicates whether a particular bacterial family or genus is increased or decreased in both the IgE and non-IgE CMA groups compared to the HC group. Patients with IgE CMA (*n* = 56); patients with non-IgE CMA (*n* = 67); HC (*n* = 33). The *p* values in this analysis are adjusted for the covariates sex and age in months. To identify differentially abundant taxa, we performed ANCOM-BC. For comparisons among groups, Kruskal-Wallis tests followed by Dunn’s post hoc tests with Bonferroni correcion were applied. Statistically significant differences between groups are considered when *p* < 0.05.

**Table 3:** Composition of the gut fungi is altered in infants with IgE and non-IgE CMA compared to HC

Gut – fungi			
Family	IgE vs. HC (adjusted *p* values)	non-IgE vs. HC (adjusted *p* values)	
Cryptococcaceae	0.8146	0.0160	↓
Saccharomycetales_fam_Incertae_sedis	0.2266	0.0495	↓

Genus	IgE vs. HC (adjusted *p* values)	non-IgE vs. HC (adjusted *p* values)	

*Candida*	0.3146	0.0764	↓
*Cryptococcus*	0.8098	0.0281	↓

↑↓ indicates whether a particular fungal family or genus is increased or decreased in both the IgE and non-IgE CMA groups compared to the HC. The *p* values in this analysis are adjusted for sex and age in months. Patients with IgE CMA (*n* = 32); patients with non-IgE CMA (*n* = 40); HC (*n* = 28). To identify differentially abundant taxa, we performed ANCOM-BC. The *p* values in this analysis are adjusted for sex and age in months. For comparisons among groups, Kruskal-Wallis tests followed by Dunn’s post hoc tests with Bonferroni correction were applied. Statistically significant differences between groups are considered when *p* < 0.05.

### Compared to non-IgE CMA patients and healthy controls, the changes in the composition of the cheek microbiota of IgE CMA patients were more pronounced than in the gut microbiota

**Figure 3:**
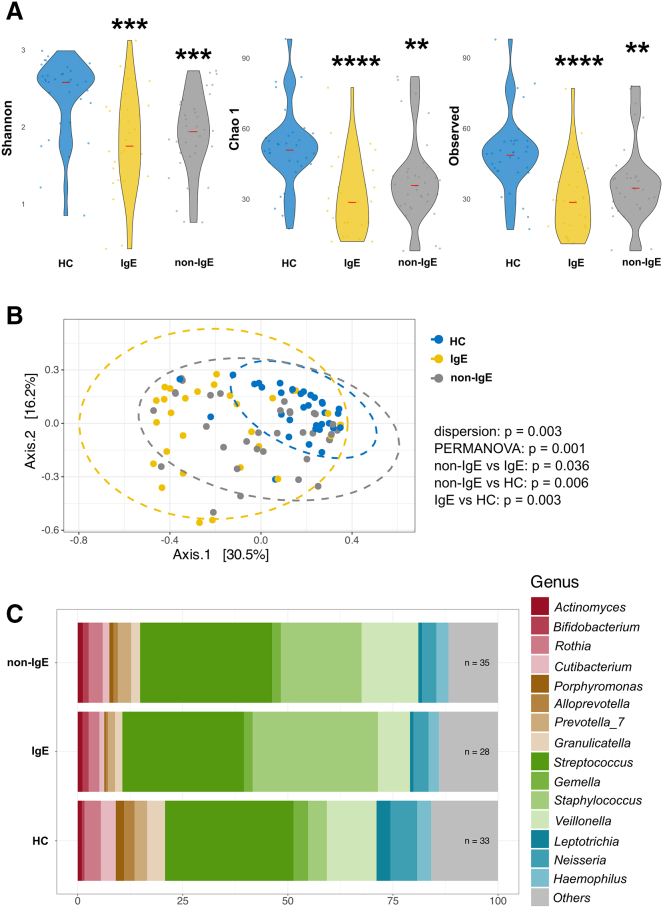
Skin bacteria in the cheek of patients with non-IgE CMA differ from patients with IgE CMA and HC (A) Alpha diversity expressed as Shannon diversity index, Chao1, observed taxa. The significant differences among groups were tested using the Kruskal-Wallis test followed by Dunn’s test with Bonferroni correction. (B) Beta diversity was calculated using Bray-Curtis dissimilarities and visualized by PCoA where each point represents one patient or healthy control and tested with PERMANOVA. (C) Abundances of the 15 most abundant bacterial genera. These data display results without covariates for age and sex. Statistically significant differences between groups are marked with ∗ (∗*p* < 0.05, ∗∗*p* < 0.01, ∗∗∗*p* < 0.001, ∗∗∗∗*p* < 0.0001).

The differences in the skin microbiota are related to the health status of the skin^32^; therefore, we exclusively sampled active lesional sites in the cheek and the popliteal fossa of AD patients in the IgE CMA vs. non-IgE CMA group and we compared them to HC. We found significant changes in the skin in alpha and beta diversity and at the taxonomic level in relation to the FA type (IgE CMA vs. non-IgE CMA). This difference could be seen especially in the skin microbiota of the cheek, where the skin is mostly encountering food allergens (Figure 3). We would like to highlight here, that the alpha diversity in patients with IgE CMA is the lowest compared to patients with non-IgE CMA or HC (Figure 3A). Beta diversity of IgE CMA patients also differs not only from HC but also from non-IgE CMA patients (Figure 3B). Interestingly, when looking at the skin microbiota composition in infants with AD and FA, far more skin microbiota-related changes are found in patients with IgE CMA than in non-IgE CMA compared to HC (Figures 3C and S1C); regardless of whether it is the skin microbiome of cheek or that of the popliteal fossa (Tables 4 and S1).

**Table 4:** Composition of the cheek bacteria is altered in infants with IgE and non-IgE CMA compared to HC

Skin – bacteria (cheek)				
Family	IgE vs. HC (adjusted *p* values)		non-IgE vs. HC (adjusted *p* values)	
Aerococcaceae	0.0001	↓	0.0241	↓
Beggiatoaceae	0.1049	↑	0.0172	
Carnobacteriaceae	0.0153	↓	0.9413	
Dermacoccaceae	0.0478	↓	0.8849	
Flavobacteriaceae	0.0455	↓	0.1722	
Gemellaceae	0.0001	↓	0.3876	
Leptotrichiaceae	0.0113	↓	0.6400	
Micrococcaceae	0.0000	↓	0.6821	
Moraxellaceae	0.0000	↓	0.0067	↓
Neisseriaceae	0.0454	↓	0.9413	
Porphyromonadaceae	0.0000	↓	0.4206	
Prevotellaceae	0.0455	↓	0.8969	
Propionibacteriaceae	0.0006	↓	0.4653	
Rhodobacteraceae	0.0757		0.0067	↓
Staphylococcaceae	0.0789	↑	0.0636	↑
Streptococcaceae	0.0016	↓	0.9413	
Veillonellaceae	0.0001	↓	0.6821	
Weeksellaceae	0.0062	↓	0.5190	

Genus	IgE vs. HC (adjusted *p* values)		non-IgE vs. HC (adjusted *p* values)	

*Abiotrophia*	0.0030	↓	0.1317	
*Alloprevotella*	0.0002	↓	0.6227	
*Bergeyella*	0.0091	↓	0.6227	
*Cutibacterium*	0.0009	↓	0.4792	
*Enhydrobacter*	0.0000	↓	0.1640	
*Gemella*	0.0001	↓	0.4647	
*Granulicatella*	0.0210	↓	0.9974	
*Kocuria*	0.0355	↓	0.6311	
*Lactobacillus*	0.0422	↑	0.6227	
*Lactococcus*	0.0105	↓	0.1314	
*Lawsonella*	0.0454	↑	0.2738	
*Leptotrichia*	0.0134	↓	0.6227	
*Micrococcus*	0.0267	↓	0.4792	
*Neisseria*	0.0391	↓	0.8577	
*Oribacterium*	0.0200	↓	0.6590	
*Paracoccus*	0.0881		0.0071	↓
*Porphyromonas*	0.0000	↓	0.4792	
*Prevotella*	0.0091	↓	0.6227	
*Rothia*	0.0001	↓	0.6234	
*Streptococcus*	0.0025	↓	0.9009	
*Veillonella*	0.0002	↓	0.8577	

↑↓ indicates whether a particular bacterial family or genus is increased or decreased in both the IgE and non-IgE CMA groups compared to the HC group. Patients with IgE CMA (*n* = 28); patients with non-IgE CMA (*n* = 35); HC (*n* = 33). To identify differentially abundant taxa, we performed ANCOM-BC. The *p* values in this analysis are adjusted for sex and age in months. For comparisons among groups, Kruskal-Wallis tests followed by Dunn’s post hoc tests with Bonferroni correction were applied. Statistically significant differences between groups are considered when *p* < 0.05.

The skin microbiota in the cheek of IgE CMA patients contained a lower abundance of the families Carnobacteriaceae, Dermacoccaceae, Flavobacteriaceae, Gemellaceae, Leptotrichiaceae, Micrococcaceae, Neisseriaceae, Porphyromonadaceae, Prevotellaceae, Propionibacteriaceae, Streptococcaceae, Veillonellaceae, and Weeksellaceae and lower abundance of the genera *Abiotrophia*, *Alloprevotella*, *Bergeyella*, *Cutibacterium*, *Enhydrobacter*, *Gemella*, *Granulicatella*, *Kocuria*, *Lactococcus*, *Leptotrichia*, *Micrococcus*, *Neisseria*, *Oribacterium*, *Porphyromonas*, *Prevotella*, *Rothia*, *Streptococcus*, and *Veillonella* compared to HC (Table 4). Patients with IgE CMA have higher abundance of the family Beggiatoaceae as well as genera *Lactobacillus* and *Lawsonella* compared to HC (Table 4). Lower abundance of the families Aerococcaceae and Moraxellaceae was found in both allergy groups when compared to HC (Table 4). Abundance of family Staphylococcaceae was found higher in both allergy groups compared to HC (Table 4). On the other hand, non-IgE CMA patients have lower abundance of the family Rhodobacteraceae and lower abundance of the genus *Paracoccus* compared to HC (Table 4). Similar results were also found in the popliteal fossa, the distal moist area where atopic lesions usually occur (Table S1). Additionally, we investigated whether there is a correlation between severity of AD and the abundance of bacterial families and genera on cheek of both study groups. We found a negative correlation between severity of AD and the abundance of the families Carnobacteriaceae, Gemellaceae, Leptotrichaceae, Micrococcaceae, Neisseriaceae, Porphyromonadaceae, Streptococcaceae, and Weeksellaceae as well as the genera *Alloprevotella*, *Bergeyella*, *Gemella*, *Granulicatella*, *Leptotrichia*, *Neisseria*, *Porphyromonas*, *Rothia*, and *Streptococcus* in AD patients with IgE CMA but not in non-IgE CMA patients (Figure S2). Next, we investigated whether there is a correlation between fungal and bacterial constituents of the gut microbiome within our study groups. Patients with IgE CMA have statistically significant positive correlation between the occurrences of genus *Klebsiella* with fungal division Chytridiomycota. HC has statistically significant positive correlation between the occurrence of genus *Klebsiella* with fungal division Malassezia. On the contrary, a negative correlation of these two taxonomical units was observed in patients with non-IgE CMA. Interestingly, the genus *Faecalibacterium* was positively correlated with genus *Filobasidium* in HC (Figure S3). Then, we investigated whether there is a correlation between the bacterial genera of the skin and gut within our study groups. In HC, we found a statistically significant positive correlation between the occurrence of genus *Anaerostipes*, the *Ruminococcus gnavus* group in intestine and the genus *Prevotella* on the skin. Interestingly, the *Eubacterium eligens* group in the gut was positively correlated with genus *Paracoccus* in skin of patients with IgE CMA, in contrast to a negative correlation of these taxa in patients with non-IgE CMA (Figure S4).

### Patients with non-IgE CMA have different levels of markers of immune responses to bacteria and of epithelial disruption than patients with IgE CMA

**Figure 4:**
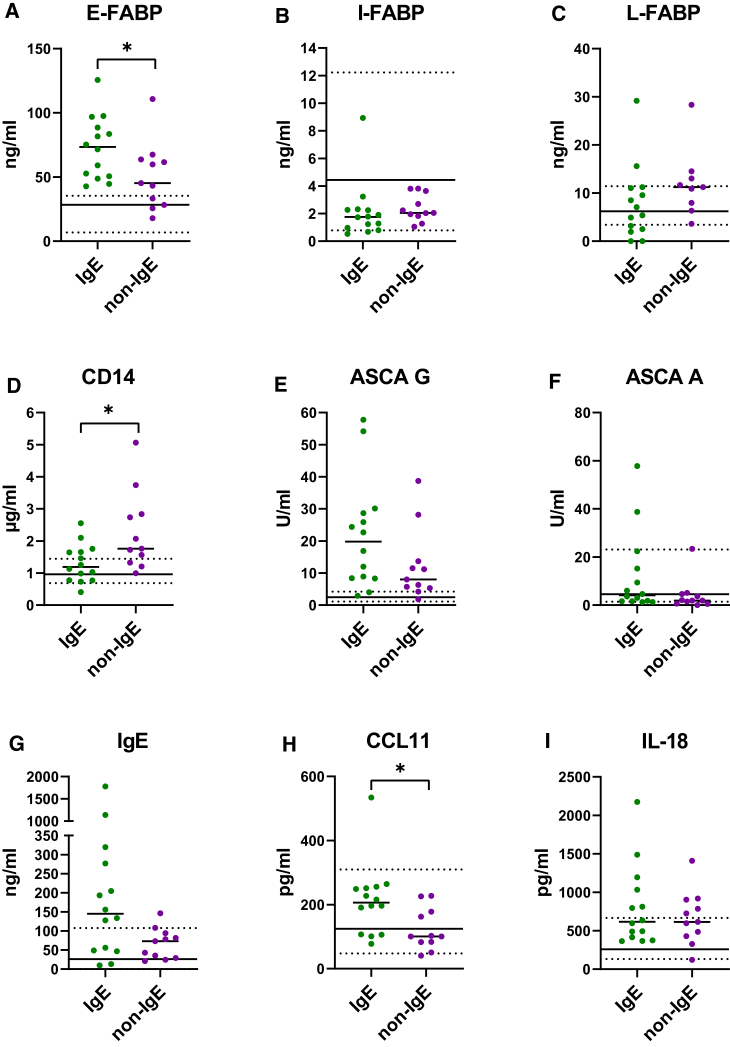
Changes in serum markers levels between IgE and non-IgE CMA patients (A–C) Molecules associated with epithelial barrier damage. (D–F) molecules involved in immune response associated with microbial translocation. (G–I) molecules regulating the immune response. The level of serum markers is depicted as the mean of values. The statistical differences between two groups were analyzed by non-parametric Mann-Whitney test. Statistically significant differences between groups are marked with ∗ (∗*p* < 0.05). Black lines depict the median (full line) and 95% CI (dotted lines) of healthy controls (HCs). IgE CMA patients (*n* = 14), non-IgE CMA patients (*n* = 11), and HC (*n* = 5–7).

Next, we carried out a detailed analysis of the serum biomarkers to identify differences in immune responses between IgE and non-IgE CMA patients with AD. From the cohort of all patients, we selected 25 for whom we were able to both characterize peripheral blood mononuclear cells (PBMCs) and determine the levels of individual serum biomarker. Unfortunately, due to ethical constraints, we were unable to collect blood samples for PBMC analysis from healthy children. However, we were able to obtain serum samples for biomarker analysis from a subset of HC, which we depict as the median and 95% confidence interval (Figure 4). The demographic and clinical characteristics of patients and HC included in this analysis are summarized in Table S2. In patients’ sera, we performed an analysis of molecules that characterize the intestinal barrier damage, immune response to bacteria and associated markers of FA or AD (Figure 4). To ensure that these changes were not related to the severity of AD, we compared the scoring of atopic dermatitis (SCORAD) values and the concentration of CCL17, an AD marker, in our study groups. We found that neither SCORAD nor CCL17 differ in IgE and non-IgE CMA patients’ cohorts (Figure S5). Regarding the markers related to the integrity of the epithelial barrier, fatty acids binding proteins (FABPs), we found a significant increase in epidermal FABP (E-FABP) levels, but no differences in intestinal FABP (I-FABP) or liver FABP (L-FABP) levels in patients with IgE CMA compared to non-IgE CMA patients (Figures 4A–4C). As for the immune response to bacteria, we found a significant increase in soluble CD14 levels in patients with non-IgE CMA compared to IgE CMA (Figure 4D). We have not found any differences in levels of anti-Saccharomyces cerevisiae antibody (ASCA) immunoglobulin G (IgG) and ASCA immunoglobulin A (IgA) between patients with IgE and non-IgE CMA (Figures 4E, 4F, and 4I). Unsurprisingly, we confirmed that patients with IgE CMA have higher IgE levels compared to non-IgE CMA patients (Figure 4G). Moreover, we found increased levels of CCL11 a chemoattractant for eosinophils and Th2 cells in patients with IgE CMA compared to non-IgE CMA patients (Figure 4H). E-FABP, CD14, and CCL11 discriminated IgE and non-IgE CMA patients areas under the curve (AUCs) of 0.74 (95% CI 0.60–0.88), 0.65 (0.49–0.80), and 0.76 (0.62–0.91), respectively (Figure S6). Compared to HC, E-FABP, and ASCA IgG in both patient groups, CD14 in non-CMA and IgE in CMA patients were upregulated. Our results suggest that patients with non-IgE CMA have a stronger immune response to microbial translocation which is confirmed here by the production of CD14.

### Patients with a non-IgE CMA have a higher regulatory response and a more pronounced immune response to fungi than patients with an IgE CMA

**Figure 5:**
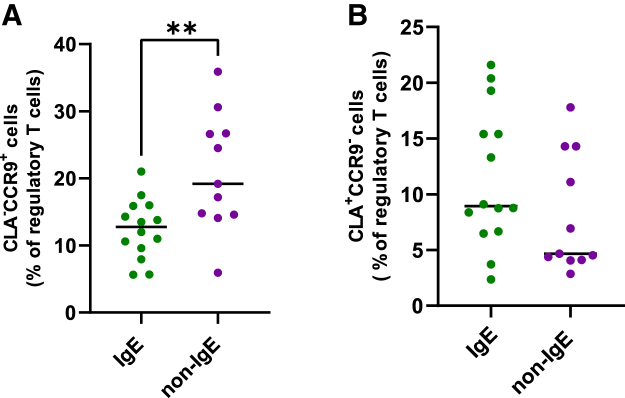
Patients with non-IgE CMA have more regulatory T lymphocytes homing to the gut than patients with IgE CMA (A) Phenotypic profile of unstimulated circulating regulatory T lymphocytes homing to gut (CD3^+^CD4^+^CD25^+^FoxP3^+^CLA^−^CCR9^+^). (B) Phenotypic profile of unstimulated circulating regulatory T lymphocytes homing to skin (CD3^+^CD4^+^CD25^+^FoxP3^+^CLA^+^CCR9^−^). Statistically significant differences between groups are determined with *t* test (∗∗*p* < 0.01). IgE CMA patients (*n* = 14); non-IgE CMA patients (*n* = 11).

**Figure 6:**
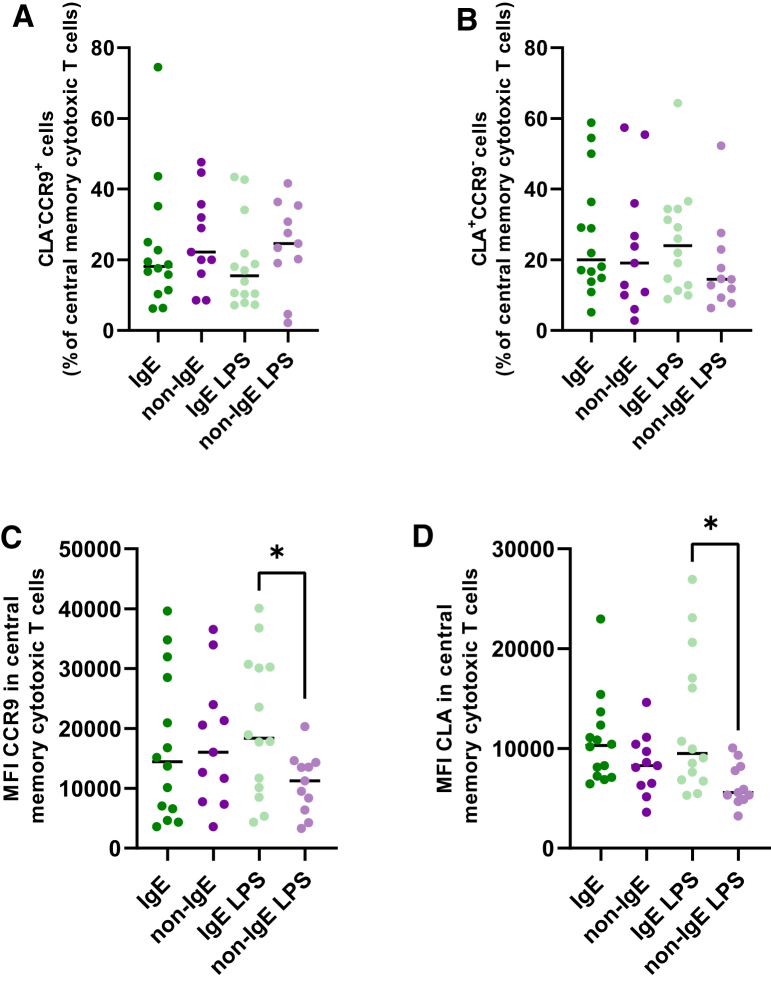
Patients with non-IgE CMA have a less pronounced homing to the gut and the skin on central memory T cytotoxic lymphocytes upon stimulation by LPS compared to IgE CMA patients (A–D) Phenotypic profile of circulating CD8^+^CD45RO^+^CCR7^+^ T lymphocytes homing to (A) gut (CCR9^+^) or to (B) skin (CLA^+^) in LPS stimulated and unstimulated cells. Expression of surface markers of CD8^+^CD45RO^+^CCR7^+^ T lymphocytes homing to (C) gut (CCR9^+^) or to (D) skin (CLA^+^) in LPS stimulated and unstimulated cells. Mean fluorescence index (MFI) for each of the indicated markers (CLA^+^ or CCR9^+^) was evaluated. Statistically significant differences between groups were evaluated by *t* test (∗*p* < 0.05). IgE (*n* = 14); non-IgE (*n* = 11); LPS, PBMC stimulated with LPS.

**Figure 7:**
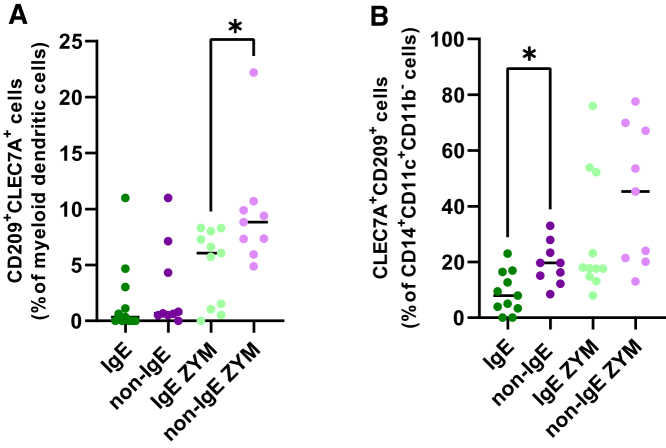
Patients with a non-IgE CMA have an enhanced antifungal immune response in specific DC subsets compared to patients with an IgE CMA (A) Phenotypic profile of circulating myeloid dendritic cells expressing CLEC7A^+^CD209^+^ in unstimulated and in zymosan (ZYM) stimulated cells. (B) Phenotypic profile of CD14^+^CD11c^+^CD11b^−^CLEC7A^+^CD209^+^ in ZYM stimulated and unstimulated cells. Statistically significant differences between groups are marked with ∗ (∗*p* < 0.05). IgE CMA patients (*n* = 11); non-IgE CMA patients (*n* = 9); ZYM, PBMC stimulated with zymosan.

We continued with the analysis of PBMCs from selected patients and analyzed the differences between the T cell profiles of IgE and non-IgE CMA patients with AD. First, we analyzed the frequency of T helper cells (CD3^+^CD4^+^) and cytotoxic T cells (CD3^+^CD8^+^) populations in experimental groups. We found no significant differences in the abundance of these two populations between the IgE and non-IgE CMA groups (Figure S7). Then, we assessed the expression of homing molecules to the gut or skin barrier as well as T cells phenotypes as shown in our gating strategy (Figure S8). We have not found any differences in T regulatory lymphocytes (Tregs) between the IgE and non-IgE CMA patients (Figure S9). Figure 5A illustrates that patients with non-IgE CMA have more Tregs homing to gut (CD3^+^CD4^+^CD25^+^FoxP3^+^CLA^−^CCR9^+^) compared to IgE CMA patients. There were no significant differences between the groups in frequency of Tregs homing to skin (CD3^+^CD4^+^CD25^+^FoxP3^+^CLA^+^CCR9^−^) (Figure 5B). These data suggest a greater targeting of Treg cells to the gut in individuals with non-IgE CMA, which has no effect on the frequency of Tregs homing to skin. Given that non-IgE CMA patients have a significantly elevated level of soluble CD14 connected to the immune response to bacteria, we were looking at their cytotoxic T cells’ response upon bacterial antigens (e.g., lipopolysaccharide [LPS]) and whether these activated cells are homing to the gut or skin. We evaluated the functional profile of central memory CD8^+^CD45RO^+^CCR7^+^ T lymphocytes homing to skin (CLA^+^) or to the gut (CCR9^+^) upon LPS stimulation. CCR7^+^CD45RO^+^ memory T cells have no direct effector function but efficiently stimulate dendritic cells (DC) and differentiate into CCR7^−^CD45RO^+^ effector memory T cells upon secondary stimulation. We found no differences in the frequency of CD8^+^CD45RO^+^CCR7^+^ T lymphocytes homing to the skin (CLA^+^) or to the gut (CCR9^+^) (Figures 6A and 6B), but we found a lower mean fluorescence intensity upon LPS stimulation in the non-IgE CMA group compared to IgE CMA patients (Figures 6C and 6D). Furthermore, we found no significant differences in CD45RO^+^CCR7^−^ effector memory T cells in unstimulated cells or in LPS-stimulated cells (Figure S10). When analyzing the role of myeloid DC in the response to fungi, we observed an increased expression of CLEC7A^+^CD209^+^ in myeloid DC of non-IgE CMA patients after stimulation by zymosan (Figure 7A). Interestingly, we observed significant increase in CD14^+^CD11c^+^CD11b^−^CLEC7A^+^CD209^+^ in non-stimulated cells of non-IgE CMA patients (Figure 7B).

## Discussion

Cutaneous sensitization most likely leads to the induction of IgE mediated-FA in the majority of cases. In contrast, clinical manifestations of non-IgE-mediated FA are predominantly restricted to gastrointestinal symptoms, suggesting that the underlying immune mechanisms could be more strongly influenced by the intestinal rather than the cutaneous microbiome. However, there is growing evidence that inflammation in the skin drives intestinal remodeling via circulating inflammatory signals, microbiome alterations, metabolites, and the nervous system.^15^ Therefore, we investigated the markers associated with changes in the microbiota and immune responses within the gut-skin axis of IgE and non-IgE CMA patients with AD to characterize the specifics of the disease endotypes.

The relationship between the gut microbiome and allergic diseases has been frequently investigated, and microbial signatures specific to the different allergies have been identified, although there is no general consensus and the causality remains uncertain.^22^^,^^23^^,^^28^^,^^33^ While the mechanisms by which microbiota contributes to the pathogenesis of AD is the subject of extensive research, the way in which skin microbiota progresses to gastrointestinal disease is not yet clear. The novelty of our study is that we analyzed not only gut bacteria but also gut fungi and skin bacteria in patients with IgE and non-IgE CMA that were linked to the immune response.

Recent research indicates that infants with CMA often exhibit alterations in gut microbiota composition and diversity. While some studies report significantly lower alpha diversity in CMA patients, others find differences in specific microbial taxa abundances without significant changes in overall diversity indices.^22^^,^^34^^,^^35^ Our findings contribute to clarifying this issue by demonstrating that patients with non-IgE CMA exhibit even lower alpha diversity compared to IgE CMA patients in their gut microbiota. In a Spanish cohort of infants with CMA, more Bifidobacteriaceae and Streptococcaceae family were found in stool samples from HC than in infants with CMA.^33^ The occurrence of family Bifidobacteriaceae also differed when the type of feeding was considered. Higher levels of family Bifidobacteriaceae were present in patients fed with breast milk than in patients fed with extensively hydrolyzed milk formula.^33^ As the patients in our study were almost all breastfed at the time of sampling, we suspect that this may be the reason why we have found no differences in the presence of family Bifidobacteriaceae in the gut. Here, we demonstrated a lower abundance of family Streptococcaceae and a significantly lower abundance of the genus *Streptococcus* in gut of patients with non-IgE CMA but not in IgE CMA patients compared to HC. Interestingly, IgE CMA patients, but not non-IgE CMA patients, showed significantly higher abundance of family Streptococcaceae and the genus *Streptococcus* on skin of cheek compared to HC. Since we are aware that streptococci are classified as common commensals and that we were unable to assign the increased genus *Streptococcus* at the species level, we can only speculate here, whether this genus is a producer of specific toxins that worsen the course of AD,^36^ or whether there is a struggle for a niche via the production of antimicrobial peptides that favors the growth of specific streptococci on diseased skin.^37^ Rather, this finding emphasizes the importance of the presence of streptococci in the skin or gut in the pathogenesis of AD and IgE or non-IgE CMA. Additionally, infants with AD and IgE or non-IgE CMA react differently to certain probiotic strains supplementation.^38^ Cukrowska et al. showed that administration of probiotic comprising *Lactobacillus rhamnosus* ŁOCK 0900, *Lactobacillus rhamnosus* ŁOCK 0908, and *Lactobacillus casei* ŁOCK 0918 resulted in SCORAD improvement mainly in allergen sensitized patients compared to those without IgE sensitization.^38^ Moreover, the gut microbiome of CMA children who developed tolerance to allergen did not differ significantly from that of children who still had CMA suggesting that the structure of the gut microbiome of CMA children is not fully restored despite the development of oral tolerance.^39^

Our study describes the relationship between skin microbiota composition in patients with AD and CMA. We found not only decreased alpha and differences in beta diversity in the skin microbiota of IgE and non-IgE CMA patients compared to HC but also differences in beta diversity between those two types of CMA. In addition, we found pronounced changes in the skin microbiome of IgE CMA patients in the form of a significant decrease in several bacterial genera and families. Similarly, we have previously shown that the skin microbiota of patients with different types of IBD differs significantly from that of healthy individuals. This difference is more pronounced in patients with Crohn’s disease compared to patients with ulcerative colitis.^40^ Interestingly, Leung et al. utilized skin tape stripping for complex multi-omics analysis and identified potential biomarkers indicating a specific stratum corneum defect in children with AD and peanut FA compared to those with AD alone. However, they found no changes in the composition of the skin microbiota in patients with AD and peanut FA compared to patients with AD alone. Contrary to our study, their analysis of the skin microbiota was performed on the upper extremities and not on the cheek, where direct contact with the allergen could trigger a different immune response leading to corresponding changes in the skin microbiome.^41^

The mycobiome is another source of microbial signals capable of directing maladaptive immune imprinting, particularly during early life. Mycobiome perturbations can lead to Th2 skewing and overt allergic responses in the gut and far-off mucosal sites.^42^ Interactions between bacteria and fungi in the gut may have important implications for the dynamics of the succession community in the gut of infants as well as for the known time-dependent interactions between the immune system and the bacterial microbiota, which ultimately determine the immune calibration relevant for the course of allergic diseases.^43^^,^^44^ Fungal dysbiosis is associated with the occurrence of AD,^42^ but not much is known about its role in FA. Our study shows for the first time that patients with non-IgE CMA have lower abundance of the genera *Cryptococcus* and *Candida*. This finding is quite surprising since the overgrowth of *Cryptococcus neoformans* is usually connected to IBD patients using immunomodulators or increased infections in immunocompromised patients.^45^^,^^46^ Taken together, the picture of gut and skin microbiota differs in both types of CMA. Far more pronounced changes compared to HC in the composition of the skin microbiome can be observed in patients with IgE allergy regardless of the severity of AD. It seems that our study cohort of IgE CMA patients possesses specific taxa whose higher abundance is associated with milder AD symptoms, indicating that their presence may be protective or linked to more balanced immune regulation. Similarly, loss of beneficial skin bacteria (e.g., genera *Streptococcus*, *Rothia*, and *Granulicatella*) mirrors the lower bacterial diversity and creates an environment that promotes skin barrier dysfunction and type 2 inflammation, which in turn increases the risk of IgE-mediated CMA. This may indicate that patients with IgE CMA may have a unique endotype of AD connected to specific skin microbiota composition.^47^ Subsequently, we investigated additional contributors to allergic manifestation that may serve as early serum biomarkers capable of distinguishing between the distinct pathogenic mechanisms underlying IgE- and non-IgE-mediated allergy, or alternatively, to differentiate phenotypic variants of AD. First, we analyzed markers associated with epithelial barrier integrity. We found no changes in serum levels of I-FABP, and L-FABP between the groups but elevated levels of E-FABP in patients with IgE CMA. There is a clear link between fatty acid-binding protein, E-FABP, and AD since both acute and chronic AD lesions express significantly more E-FABP than HC both in skin and in serum.^48^ Moreover, E-FABP was proved as potential prognostic marker of atopic march.^48^ This is consistent with our results, as we described increased E-FABP serum level in IgE CMA patients compared to the non-IgE CMA patients. Suggesting E-FABP in serum could serve as a potential biomarker to predict high-risk individuals for atopic march progression.

Further, we analyzed serum markers related to the microbiota or allergic immune response. Earlier studies showed a positive correlation between CD14 gene polymorphism and allergic diseases.^49^^,^^50^ Our study is in agreement with findings of this study in which CD14 gene polymorphism was associated with an increase in serum CD14 levels and a decrease in serum IgE levels in patients with allergic diseases such as asthma and allergic rhinitis.^49^^,^^50^ This relationship in gene polymorphism of CD14 was also found in AD.^51^ Elevated soluble CD14 in patients with non-IgE allergy could be connected to increased intestinal permeability and point out the importance in the immune response outcome associated with microbial translocation as we have previously shown in patients with IBD.^52^ Additionally, since CCL11 (eotaxin) is an important chemoattractant for eosinophils and Th2 cells,^53^ it is not surprising that we found its elevated levels in patients with IgE CMA, as compared to non-IgE CMA.

To further characterize the endotypes of IgE and non-IgE CMA patients with AD, we analyzed the proportions of DC and T cells, focusing on their homing patterns in the gut and skin and on their regulatory or cytotoxic phenotypes. The critical role of commensal microbiota-induced Tregs in promoting oral tolerance and protecting against IgE-mediated food allergies has been well documented.^54^ On the other hand, the importance of non-IgE-associated microbial dysbiosis for Treg induction and the resulting immune tolerance is still poorly understood.^55^ Therefore, we analyzed the proportion of Tregs migrating into the gut and skin of patients with AD and CMA.

Gut-resident Foxp3^+^ Treg cells operate during homeostasis to establish and maintain a tolerogenic environment in the intestine. They are able to specifically sense inflammatory signals, which lead to their activation and heightening of their suppressive capacity to counteract, e.g., inflammation and inflammation-driven tissue damage.^56^^,^^57^ Their crucial suppressive capacity has been determined in control of aberrant Th2 cell responses.^58^ Perezabad et al. described that patients with an IgE allergy to milk could generally have lower number of Tregs compared to HC.^59^ Interestingly, transplantation of fecal microbiota from infant with IgE CMA to mice resulted in significant lower abundance in the accumulation and function of intestinal Treg cells which is in accordance with our findings.^55^ In addition, children who had outgrown their CMA showed a higher frequency of circulating Tregs and a lower *in vitro* proliferative response to specific milk allergens compared to children who remained allergic.^60^ Schreffler et al. showed that a higher frequency of allergen-specific Tregs has been associated with a mild clinical phenotype and a favorable prognosis in milk-allergic children.^61^ Another study has shown that newborns with reduced functional Tregs are associated with the later development of egg allergy in childhood.^62^ Here, we found that the frequency of Tregs migrating into the gut was significantly higher in non-IgE CMA patients, who had low levels of Th2-related molecules (IgE and CCL11), reduced barrier function (represented by lower levels of E-FABP) and associated with increased microbiota stimulation (here determined by higher level of CD14). We suggest that the higher levels of CCR9 positive Tregs in non-IgE CMA infant patients compared to IgE CMA patients describes not quiescence but active, microbiota-driven immune regulation. This feature may represent an immunological tug-of-war, which could explain why non-IgE-mediated CMA rarely progresses to systemic anaphylaxis yet is characterized by persistent gastrointestinal symptoms. Interestingly, recent work in mice shows that microbiota of IgE CMA infant patients, with enriched abundance of Gram-negative bacteria, induced expression of serum amyloid A1 and Th2-related genes, as well as expanded populations of Th17 and regulatory T cells dependent on TLR4 signaling.^63^ In addition, metagenomic sequencing shows that the CMA microbiota has an increased abundance of LPS biosynthesis genes,^63^ which is consistent with our finding that IgE CMA patients have higher LPS-induced mean fluorescence intensity on central memory T cytotoxic lymphocytes upon LPS stimulation. Given that microbiota-dependent peripheral RORγt^+^ Tregs exhibit Th2-suppressive potential at mucosal sites,^64^ we suppose their contributing role in regulatory mechanisms of non-IgE CMA allergic diseases. Due to no differences in basal levels of CD4^+^ or even CD4^+^CD25^+^FoxP3^+^ cells in peripheral blood of both groups of patients, we could only speculate that Treg cells expressing gut homing receptor CCR9 downregulate FoxP3 upon migration to the epithelium and convert to intraepithelial (IEL) CD4^+^ T cells in order to control intestinal immune reaction, as was shown previously.^65^

Next, we hypothesized that patients with AD and different clinical endotypes of CMA would exhibit distinct proportions of skin-homing Tregs thereby allowing better discrimination between FA and AD endotypes. A previous study described that in infants with peanut allergy, a greater proportion of peanut-specific T effector cells from the peripheral blood expressed the skin homing receptor than the gut homing receptor compared to non-allergic controls. This may indicate that the first encounter with the antigen in these cells took place in the lymph nodes of the skin and not in the Peyer’s patches in the intestine.^66^ However, our data did not support this hypothesis, as the proportion of skin-homing Tregs was comparable in AD patients with IgE-mediated and non-IgE-mediated CMA.

Resident memory T cells have no direct effector function but could efficiently stimulate DCs and differentiate into effector memory T cells upon allergen re-exposure. In our study, we found no differences in amount of cytotoxic memory T cells expressing homing receptors to gut and skin determined in blood of IgE and non-IgE CMA patients. On the other hand, we observed lower expression of gut and skin homing receptors upon stimulation by bacterial antigen LPS in these cells from non-IgE patients. The recruitment of antigen-specific T lymphocytes to the intestinal mucosa is central to the development of an effective mucosal immune response, yet the mechanism by which this process occurs remains to be fully defined. It remains uncertain what signals would selectively downregulate or drive the CCR9 expression on CD4^+^ and CD8^+^ lineage T cells. Interestingly, CCR9^-^deficiency was found to impair the recruitment of CD8^+^ T cells into small intestine intraepithelial tissues and to hamper the establishment of oral immune tolerance highlighting that CCR9 provides critical cues for the recruitment and tissue-specific function of CD8^+^ T cells.^67^

Myeloid DC can polarize Th1 and Th2 response and play an important role in immune tolerance induction.^68^^,^^69^ With the help of various C-type lectin receptors such as fungal CLEC7A, myeloid DC can recognize, internalize, and present a range of allergens from different sources, leading to sensitization.^70^ The role of fungal antigens in patients with allergies is particularly interesting, as fungi are important allergens and contain many immunogens on their surface structures. Single nucleotide polymorphisms in the genes encoding the fungal recognition receptors CLEC7A and mannose-binding lectin have been associated with susceptibility to allergic asthma.^71^ Moreover, mycobiome dysbiosis in early childhood can contribute to a maladaptive immune imprint that favors atopic diseases.^42^ In this context, it is also important to note that the composition of fungal mycobiota changes dramatically during the first year of life and that early behavioral or therapeutic interventions have the potential to alter the fungal communities in the gut of infants, with implications for the long-term health of the child.^72^ Here, we showed that expression of CLEC7A^+^CD209^+^ in myeloid DC is elevated upon stimulation by zymosan in patients with non-IgE CMA compared to IgE CMA patients. The same pattern is also found in other DC types (CD14^+^CD11c^+^CD11b^−^CLEC7A^+^CD209^+^) and is even more pronounced in non-stimulated cells of patients with non-IgE CMA. Since antifungal therapy ameliorates colitis in Clec7a knockout mice and CLEC7A is associated with the severity of UC in humans, the role of this receptor in patients with non-IgE CMA may play a role in protecting against fungal-induced intestinal inflammation.^73^ Nonetheless, future work is needed to validate our results in animal models, to establish a causal role for early childhood intestinal fungal dysbiosis and to search for related mechanisms in allergic outcomes.

Taken together, we identified a distinct bacterial pattern, primarily composed of skin associated taxa, which may determine the host to an IgE and non-IgE allergic response to milk in patients with AD. Further, we have identified serum biomarkers associated with epithelial barrier dysfunction and immune response to microbiota that can help distinguish between IgE and non-IgE CMA endotypes in infants with AD. We show that immune system of AD patients with non-IgE CMA demonstrates heightened responsiveness to microbial stimuli, as evidenced by increased serum CD14 levels and more robust immune responses to fungal antigens. A detailed characterization of allergic endotypes has become essential for the development of personalized treatments, as each subtype may respond differently to immunomodulatory therapies. However, further analysis is needed to describe the direct influence of the presence of AD in the pathogenesis of FA or to describe the endotype of AD resulting from FA.

### Limitations of the study

Limitations of this study include the use of 16S rRNA/ITS1-based sequencing methods, which are often insufficient to identify bacteria to species level. It is known that different species of the same genus can have very different effects in the gut/on the skin, making it difficult to hypothesize the biological mechanisms underlying these results. Another technical limitation is that only stool samples were collected and analyzed, although many of the interactions between the gut microbiota and the intestinal mucosa and immune system take place in the small intestine. However, it would have been unethical to invasively obtain samples from infants in our study. Another limitation of this study is that not enough blood samples from healthy children were collected for serum and PBMC analysis as a control. We considered it unethical to take blood from healthy children without a reason, or/and mothers of healthy children are not in favor of these blood draws. Next, we are aware that some of our conclusions are rather speculative due to the small number of patients analyzed, but we are convinced that they have great medical potential. However, these associations need to be validated by rigorous experimental studies and replication in independent infant populations.

## Resource availability

### Lead contact

Further information and requests for resources and reagents should be directed to and will be fulfilled by the lead contact, Zuzana Jiraskova Zakostelska (zakostelska@biomed.cas.cz).

### Materials availability

This study did not generate new unique materials/reagents.

### Data and code availability

• The raw sequence data are available in the Sequence Read Archive (SRA) under the BioProject: PRJNA1213719.
• This study generated no custom code.
• For any additional information, please contact the lead contact, Zuzana Jiraskova Zakostelska (zakostelska@biomed.cas.cz).

## Acknowledgments

The authors thank Alena Kubatova and Jan Svoboda for excellent technical assistance and Martin Mihula for sample preparation. The authors are grateful to all the families who participated in this study, and the nurses and laboratory technicians from Motol University Hospital. The authors acknowledge the Cytometry and Microscopy Facility at the Institute of Microbiology of the CAS, Prague, CZ, for the use of cytometry equipment. The authors acknowledge the CF Genomics of CEITEC supported by the NCMG research infrastructure (LM2023067 funded by MEYS CR) for their support with obtaining scientific data presented in this paper. This work was supported by grants NU20-05-00038 from the 10.13039/501100009553Czech Health Research Council and grant Talking microbes - understanding microbial interactions within One Health framework (CZ.02.01.01/00/22_008/0004597) from the Ministry of Education, Youth and Sports of the Czech Republic, and the 10.13039/501100004240Academy of Sciences of the Czech Republic (LQ200202105). L.B. was supported by MUNI/A/1685/2024 grant.

## Author contributions

Z.J.Z., H.T.-H., D.S., E.K., and A.P. conceived and designed the research; T.T., Z.J.Z., and D.S. wrote the manuscript; E.K., A.P., S.Capkova, and A.S. examined the patients and healthy controls and collected samples; V.B., L.B., S.Coufal, and J.K. performed the analysis of the sequencing data and statistics; Z.J.Z., M.K., T.T., F.R., T.S., and D.S. helped carry out the experiments and analyzed and interpreted the data. All authors revised and approved the final version of the manuscript.

## Declaration of interests

The authors declare that the research was conducted in the absence of any commercial or financial relationships that could be construed as a potential conflict of interest.

## STAR★Methods

### Key resources table

**Table:** 

REAGENT or RESOURCE	SOURCE	IDENTIFIER
**Antibodies**		

CD3, Alexa Fluor™ 488, eBioscience™	Thermo Fisher Scientific, Invitrogen	Cat#53-0037-42; RRID:AB_1907370; clone OKT3
CD4, violetFluor™ 450™	Cell Signaling Technology	Cat#26755S; RRID:AB_2798930; clone RPA-T4
CD8, redFluor™ 710	Abcam	Cat#ab241937; clone RPA-T8
CD25, PE-eFluor™ 610, eBioscience™	Thermo Fisher Scientific, Invitrogen	Cat#61-0257-42; RRID:AB_2574544; clone CD25-4E3
FoxP3, APC, eBioscience™	Thermo Fisher Scientific, Invitrogen	Cat#17-4777-42; RRID:AB_10804651; clone 236A/E7
CCR7, PerCP-eFluor™ 710, eBioscience™	Thermo Fisher Scientific, Invitrogen	Cat#46-1979-42; RRID:AB_10853814; clone 3D12
CD45RA, StarBright Violet 710	Bio-Rad	Cat#MCA88SBV710; RRID:AB_3101672; clone F8-11-13
CD45RO, Super Bright™ 600, eBioscience™	Thermo Fisher Scientific, Invitrogen	Cat#63-0457-42; RRID:AB_2662466; clone UCHL1
CCR9, Brilliant Violet 510	BD Biosciences	Cat#752588; RRID:AB_2917575; clone C9Mab-1
CLA, PE/Cyanine7	BioLegend	Cat#321316; RRID:AB_2565768; clone HECA-452
CD14, APC-eFluor™ 780, eBioscience™	Thermo Fisher Scientific, Invitrogen	Cat#47-0149-41; RRID:AB_1834359; clone 61D3
CD16, APC-eFluor™ 780, eBioscience™	Thermo Fisher Scientific, Invitrogen	Cat#47-0166-42; RRID:AB_2848353; clone 3G8
CD19, APC-eFluor™ 780, eBioscience™	Thermo Fisher Scientific, Invitrogen	Cat#47-0199-42; RRID:AB_1582230; clone HIB19
CD56, APC-eFluor™ 780, eBioscience™	Thermo Fisher Scientific, Invitrogen	Cat#47-0567-42; RRID:AB_10854573; clone CMSSB
CD11c, Fluorescein isothiocyanate, eBioscience (FITC)	Thermo Fisher Scientific, Invitrogen	Cat#11-0116-42; RRID:AB_10718106; clone 45903
HLA-DR, eFluor™ 506, eBioscience™	Thermo Fisher Scientific, Invitrogen	Cat#69-9956-41; RRID:AB_2762583; clone LN3
CD123, PE, eBioscience™	Thermo Fisher Scientific, Invitrogen	Cat#12-1239-42, RRID:AB_10609206; clone 6H6
CD11b, Alexa Fluor® 700	BioLegend	Cat#301356; RRID:AB_2750075; clone ICRF44
CD209, Brilliant Violet 421™	BioLegend	Cat#330118; RRID:AB_2734324; clone 9E9A8
CLEC7A, PerCP-eFluor™ 710, eBioscience™	Thermo Fisher Scientific, Invitrogen	Cat#46-9856-42; RRID:AB_2573910; clone 1500
CD3, APC-eFluor™ 780, eBioscience™	Thermo Fisher Scientific, Invitrogen	Cat#47-0038-42; RRID:AB_1272042; clone UCHT1
CD14, Brilliant Violet 605™	BioLegend	Cat#301834; RRID:AB_2563798; clone M5E2
Fc Receptor Binding Inhibitor Polyclonal Antibody, eBioscience™	Thermo Fisher Scientific, Invitrogen	Cat#14-9161-73; RRID:AB_468582

**Biological samples**		

blood samples	this study	
stool samples	this study	
ski swabs	this study	
ZymoBIOMICS Microbial Community Standard (linear)	Zymo Research	Cat#D6300
ZymoBIOMICS Microbial Community Standard (log distribution)	Zymo Research	Cat#D6310
ZymoBIOMICS Microbial Community DNA Standard (linear)	Zymo Research	Cat#D6305
ZymoBIOMICS Microbial Community DNA Standard (log distribution)	Zymo Research	Cat#D6311

**Chemicals, peptides, and recombinant proteins**		

KAPA HiFi HotStart Ready Mix	Roche	Cat#07958935001
Ficoll-Paque™ PLUS density gradient media	Citiva	Cat#17144002
RPMI-1640 Medium (Sigma-Aldrich)	Merck KGaA	Cat#R0883
Antibiotic Antimycotic Solution (100×), Stabilized	Merck KGaA	Cat#A5955
L-Glutamine	Merck KGaA	Cat#G6392
Zymosan	InvivoGen	Cat#tlrl-zyn
LPS from Salmonella abortus equi S-form (TLRGRADE®) (Ready-to-Use)	Enzo Life Sciences	Cat#ALX-581-009
Fixable Viability Dye eFluor™ 780	Thermo Fisher Scientific, Invitrogen™, eBioscience™	Cat#65-0865-18
Foxp3 / Transcription Factor Staining Buffer Set	Thermo Fisher Scientific, Invitrogen™, eBioscience™	Cat#00-5523-00

**Critical commercial assays**		

DNeasy PowerBiofilm kit	Qiagen	Cat#24000-50
ZymoBIOMICS DNA Miniprep Kit	Zymo Research	Cat#D4300
SequalPrep™ Normalisation Plate Kit	Thermo Fisher Scientific	Cat#A1051001
DNA Clean & Concentrator Kit	Zymo Research	Cat#D4080
KAPA HyperPlus kit	Roche	Cat#07962401001
ASCA-IgA (Saccharomyces cerevisiae) ELISA kit	AESKU.GROUP	Cat#3507
ASCA-IgG (Saccharomyces cerevisiae) ELISA kit	AESKU.GROUP	Cat#3508
Human FABP5/E-FABP ELISA Kit (Colorimetric)	Novus Biologicals	Cat#NBP2-82538
Human FABP2/I-FABP DuoSet ELISA	R&D systems	Cat#DY3078
L-FABP, Human, ELISA kit	Hycult Biotech	Cat#HK404-02
Human Total IL-18 DuoSet ELISA	R&D systems	Cat#DY318-05
Human CD14 DuoSet ELISA	R&D systems	Cat#DY383
Human CCL11/Eotaxin DuoSet ELISA	R&D systems	Cat#DY320
Human IgE Uncoated ELISA Kit	Thermo Fisher Scientific, Invitrogen	Cat#88-50610-88

**Deposited data**		

BioProject	this study	PRJNA1213719

**Oligonucleotides**		

16S rRNA gene V3–V4 forward primer (341F): GTCCTACGGGNGGCWGCAG	Generi Biotech	custom made
16S rRNA gene V3–V4 reverse primer (806R): GGACTACHVGGGTWTCTAAT	Generi Biotech	custom made
Fungal ITS1 forward primer: GTAAAAGTCGTAACAAGGTTTC	Generi Biotech	custom made
Fungal ITS1 reverse primer: AAGTTCAAAGAYTCGATGATTCAC	Generi Biotech	custom made

**Software and algorithms**		

GraphPad Prism, version 9.1.2	GraftPad Software	https://www.graphpad.com
R, version 4.4.1	R Project	https://www.r-project.org/
FlowJo, version 10.1.0.0	BD	https://www.flowjo.com
ROCR	R package (CRAN)	https://cran.r-project.org/package=ROCR
Cutadapt	Martin, M. (2011) (78)	https://cutadapt.readthedocs.io/
DADA2, version 1.26.0	R package (Bioconductor)	https://benjjneb.github.io/dada2/
phyloseq, version 1.48.0	R package (Bioconductor)	https://joey711.github.io/phyloseq/
vegan, version 2.6-8	R package (CRAN)	https://cran.r-project.org/package=vegan
ANCOM-BC, version 2.8.0	R package (Bioconductor)	https://github.com/FrederickHuangLin/ANCOM-BC
zCompositions, version 1.5.0.4	R package (CRAN)	https://cran.r-project.org/package=zCompositions
coin, version 1.4-3	R package (CRAN)	https://cran.r-project.org/package=coin
rstatix, version 0.7.2	R package (CRAN)	https://cran.r-project.org/package=rstatix
ggplot2, version 3.5.1	R package (CRAN)	https://ggplot2.tidyverse.org/
pheatmap, version 1.0.12	R package (CRAN)	https://cran.r-project.org/package=pheatmap
plotly, version 4.10.4	R package (CRAN)	https://plotly.com/r/

**Other**		

FLOQSwabs® flocked swab	COPAN Diagnostics Inc	Cat#520CS01
sterile 96-well U-well tissue culture plates	TPP	Cat# TP92097

### Experimental model and study participant details

#### Study cohort

A total of 123 children with FA and AD and 33 healthy controls (HC) were enrolled in this study, which began in 2021 and ended in 2023. Infants aged 3 to 12 months participated in this cohort study (Table 1). The children were examined and detailed information on environmental exposures and clinical outcomes was collected using a combination of questionnaires and face-to-face clinical assessments. The severity of the disease in AD patients was assessed using standard clinical criteria according to Hanifin and Rajka.^74^ The severity of AD was estimated using the SCORAD index (Severity Scoring of Atopic Dermatitis). Patients are categorized into 3 groups: mild (SCORAD < 25), moderate (SCORAD 25-50) and severe (SCORAD > 50). After taking a medical and medication history, a complete dermatological and immunological examination was carried out according to international guidelines, e.g. a skin prick test for relevant environmental and food allergens as well as specific IgE tests for cow’s milk and other food allergens. In addition, a component diagnosis (e.g. casein, beta lactoglobulin, alpha lactoglobulin) and an open provocation test were carried out to assess tolerance to food allergens and their natural development.

Children who had allergen-specific IgE level for cow’s milk equal or higher than 0.35 kUA/L or who were IgE-positive to casein, beta-lactoglobulin and alpha-lactoglobulin and had a positive elimination exposure test, were considered to have IgE CMA. Patients with non-IgE CMA were identified based on the positivity in the elimination exposure test. Patients with both IgE (n = 56) and non-IgE (n = 67) food allergies were included in the study. During recruitment, questionnaires on environmental exposures, mode of delivery, breastfeeding and general health were completed. The clinical and laboratory data as well as the baseline characteristics of all patients and controls at the time of recruitment are summarized in results section. To avoid artificial disturbance of the microbiota, the patients or HC did not have an ongoing infection or were not treated with antibiotics for at least one month prior to the collection of skin, fecal and serum samples.

#### Ethics approval and consent to participate

All study participants were of Caucasian ethnicity. This study was approved by the ethics committee of the University Hospital in Motol (approval date 22/5/2019). Informed consent was obtained from the parents at the beginning of the study.

#### Ethics statement

All study participants signed informed consent forms. This study was approved by the Ethics Committee of Motol University Hospital (approval date 22/5/2019). The children's legal representatives gave their written consent for their children to take part in this study.

### Method details

#### Sample collection

Blood and stool samples as well as skin swabs were collected from patients with AD and FA and from HC during their routine dermatological examination at Motol University Hospital (Prague, Czech Republic). Parents were instructed not to use antiseptics and not to bathe or wash the affected skin areas 24 hours prior to sampling. Skin swabs were taken from approximately 4 cm^2^ area of the cheek and the popliteal fossa using a sterile FLOQSwabs (COPAN Diagnostics Inc., Murrieta, CA, USA) soaked in sterile SCF-1 buffer as previously described.^75^ Blood samples and stool samples were collected as previously described.^30^^,^^31^ All samples were stored at −80 °C until further processing.

#### Gut and skin microbiota analysis

Extraction of total DNA from skin swabs was done using the DNeasy PowerBiofilm kit (Qiagen, Hilden, Germany) with minor modifications to the protocol as previously described.^75^ DNA was isolated from the stool sample using the ZymoBIOMICS DNA Miniprep Kit (Zymo Research, Irvine, CA, USA) according to the manufacturer’s protocol. All subsequent steps were carried out exactly as described previously.^40^^,^^76^ The V3V4 region of the 16S rRNA gene and the fungal ITS1 region covered by specific primers with barcodes (341F GTCCTACGGGNGGCWGCAG and 806R GGACTACHVGGGTWTCTAAT) and (F GTAAAAGTCGTAACAAGGTTTC and R AAGTTCAAAGAYTCGATGATTCAC), respectively, were selected as representative sequences for taxonomic identification. The amplification reaction was performed with the KAPA HiFi HotStart Ready Mix (Roche, Basel, Switzerland) as follows: first denaturation step 3 min at 95 °C, followed by 25 cycles at 95 °C for 30 s, 55 °C for 30 s, 72 °C for 30 s with a final elongation step at 72 °C for 5 min using 5 ng/μL DNA for stool samples and 33 cycles of denaturation (94 °C, 3 min), annealing (55 °C, 5 s), and extension (72 °C, 2 min) for skin swab samples.^40^^,^^76^ The PCR products were checked with QIAxcel advanced capillary electrophoresis (Qiagen). Triplicates of amplicons were pooled and normalized using the SequalPrep Normalization Plate Kit (Thermo Fisher Scientific, Waltham, MA, USA), concentrated (Eppendorf Centrifugal Vacuum Concentrator, Eppendorf, Hamburg, Germany) and purified using the DNA Clean & Concentrator Kit (Zymo Research). PCR amplification negative controls, extraction and sequencing positive controls (mock communities; ZymoBIOMICS Microbial Community Standard and ZymoBIOMICS Microbial Community DNA Standard, Zymo Research; both in linear and logarithmic form) were processed in a similar manner. Amplicon libraries were then ligated with sequencing adapters using the KAPA HyperPlus kit (Roche), pooled in equimolar concentrations and sequenced. Amplicon sequencing was performed using the MiSeq platform (Illumina, San Diego, CA, USA). The raw sequence data are available in the Sequence Read Archive (SRA) under the accession number BioProject: PRJNA1213719.

#### Analysis of serum biomarkers

The analyzed biomarkers related to gut barrier function and inflammatory response were quantified in serum using a commercial enzyme-linked immunosorbent assay (ELISA).

**Table:** The list of quantified biomarkers in serum

Biomarker	Abbreviation	Manufacturer	Cat. no.
Anti–*Saccharomyces cerevisiae* antibody IgA	ASCA IgA	AESKU.GROUP	3507
Anti–*Saccharomyces cerevisiae* antibody IgG	ASCA IgG	AESKU.GROUP	3508
Epithelial fatty acid-binding protein	E-FABP	Novus Biologicals	NBP2-82538
Intestinal fatty acid-binding protein	I-FABP	R&D systems	DY3078
Liver fatty acid-binding protein	L-FABP	Hycult Biotech	HK404-02
Interleukin-18	IL-18	R&D systems	DY318-05
Soluble CD14	CD14	R&D systems	DY383
Chemokine (C-C motif) ligand 11	CCL11	R&D systems	DY320
Chemokine (C-C motif) ligand 17	CCL17	R&D systems	DY364
Immunoglobulin E	IgE	Thermo Fisher Scientific, Invitrogen	88-50610-88

#### Analysis of peripheral blood mononuclear cells

Human peripheral blood mononuclear cells (PBMCs) were isolated from collected heparinized blood using Ficoll-Paque Plus density gradient media centrifugation (Citiva, Marlborough, MA, USA) (740 × g, 30 min, room temperature (RT) and stored at −150 °C until analysis.^77^ For thawing, cryotubes were placed in a 37 °C water bath for 8 min and then transferred to a 15 mL tube. They were diluted dropwise with 8 mL of pre-warmed RPMI medium (Merck KGaA, Darmstadt, Germany), centrifuged at 300 × g for 5 min at RT, and the supernatant was then removed. After a further washing step, the cells were counted using Trypan blue exclusion and diluted to a concentration of 5 × 10^6^ live cells/mL. These cells were then transferred to sterile 96U-well tissue culture plates (TPP, Trasadingen, Switzerland) for characterization of T lymphocytes (300 thousand of cells), or for characterization of dendritic cells (500 thousand of cells) at 100 μL per well. The culture medium used was RPMI (Merck KGaA) supplemented with 10% FCS, 1% antibiotic-antimycotic solution (Merck KGaA), and 1% L-glutamine solution (Merck KGaA). The plates were placed in a humidified incubator (37 °C, 5% CO_2_) for a 2 h incubation period prior to stimulation. Subsequently, 100 μL/well of the medium or stimulus was added, and the cells were incubated under similar conditions for another 17 h. The final concentration of stimuli per well was 50 μg/mL of zymosan (InvivoGen, San Diego, CA, USA), 0.1 μg/mL lipopolysaccharide (LPS) from *Salmonella abortus equi* S-form (Enzo Life Sciences, Farmingdale, NY, USA). After the cultivation, PBMC were incubated with human FcR receptor binding inhibitor for 30 min (Thermo Fisher Scientific). To distinguish viable and dead cells, Fixable Viability Dye-eFluor 780 was added.Then were stained with fluorochrome-conjugated antibodies recognizing extracellular epitopes specific for dendritic or T cell population, for detailed description of antibodies used please see (Tables S3 and S4). To analyze intracellular antigen transcription factor FoxP3 within the T cell subset the cells were treated with eBioscience Foxp3/Transcription Factor Staining Buffer Set (Thermo Fisher Scientific) and then stained. Data were obtained by measuring the samples on LSR II (BD Biosciences, Franklin Lakes, NJ, USA) and analyzed by the FlowJo software (v. 10.1.0.0) (BD, Ashland, OR, USA). Data are expressed as the median percentage of cells within the gated population for all PBMC experiments. An example of the gating strategies used, and a list of the antibodies used for the T cell and DC panel can be found in Figures S8 and S11, and Tables S3 and S4.

### Quantification and statistical analysis

#### Sequencing data processing and statistics

##### Preprocessing and quality control of amplicon sequences

Raw sequencing reads were demultiplexed based on unique barcodes. Adapter and primer sequences were trimmed using *Cutadapt*.^78^ Further quality filtering and trimming were performed in R (version 4.4.1) with the *DADA2* package (version 1.26.0, vegan 2.6–8).^79^ Specifically: maxN = 0: Sequences containing ambiguous bases (N) were discarded; maxEE = c(2,2): Reads with more than two expected errors were removed; truncQ = 8: Reads were truncated at the first instance of a quality score below 8; rm.phix = TRUE: Any reads matching the phiX genome were removed. Paired-end reads were subsequently merged using mergePairs in *DADA2*, and an amplicon sequence variant (ASV) table was generated with makeSequenceTable. Chimeric sequences were identified and removed using removeBimeraDenovo. For taxonomic classification, *DADA2*’s assignTaxonomy function was applied with the SILVA 138.1 database for bacteria^80^ and the UNITE (sh_general_release_dynamic_25.07.2023) database for fungi.^81^ Taxa unrelated to bacteria or fungi (e.g., chloroplast, mitochondria) were filtered out.

##### Data filtering and compositional transformations

Prior to statistical analyses, sparse taxa were removed to reduce data dimensionality. A genus was retained if it appeared in at least three samples with an absolute abundance of ≥0.3% of the least-sequenced sample. Zero counts were imputed using the Bayesian-multiplicative method (cmultRepl in *zCompositions* version 1.5.0.4).^82^ To accommodate the lower abundance of fungal taxa, the default 80% zero threshold was relaxed for fungal analyses. Remaining non-zero data were transformed with a centered log-ratio (CLR) function to address compositional constraints.

##### Alpha and beta diversity

Alpha diversity (Shannon index, Chao1, and observed richness) was computed per sample. Group comparisons were done using the Wilcoxon rank-sum test for two groups or Kruskal-Wallis followed by Dunn’s post hoc test (Bonferroni-corrected) when comparing three or more groups. Only significant *p*-values (rounded to three decimals) were displayed in plots, connected by lines to indicate differences between specific groups. Beta diversity was assessed with Bray-Curtis and Jaccard dissimilarities, visualized via Principal Coordinates Analysis (PCoA). Differences among groups were evaluated via PERMANOVA (999 permutations, *adonis2* in the *vegan* package, version 2.6–8). When relevant, pairwise PERMANOVAs with Bonferroni correction were conducted. The *betadisper* function was used to verify homogeneity of group dispersions.

##### Differential abundance testing

To identify differentially abundant taxa while accounting for covariates (age and sex), we performed an Analysis of Compositions of Microbiomes with Bias Correction (ANCOM-BC)^83^ and the results are displayed in Tables in the results sections. For individual pairwise comparisons of taxa across two groups, Wilcoxon rank-sum tests were used. For comparisons among multiple groups, Kruskal-Wallis tests followed by Dunn’s post hoc tests (Bonferroni-corrected) were applied. Multiple testing corrections (Benjamini-Hochberg) were performed given the potentially high number of bacterial or fungal taxa. Bar plots show the abundance of the most abundant taxa without accounting for the covariates (age and sex).

##### Software and packages

All analyses were carried out in R (version 4.4.1). Key packages included phyloseq (version 1.48.0) for data handling, vegan (2.6–8) for diversity metrics, ANCOM-BC (2.8.0) for differential abundance, zCompositions (1.5.0.4) for zero replacement, coin (1.4–3) for nonparametric tests, rstatix (0.7.2) for post hoc analyses, and ggplot2 (3.5.1), pheatmap (1.0.12), and plotly (4.10.4) for data visualization.

#### Statistical analysis of biomarkers and peripheral blood mononuclear cells

Statistical analysis was performed using GraphPad Prism Software (version 9.1.2; San Diego, USA). The statistical differences between two groups were analyzed by non-parametric Mann-Whitney test. Differences were considered statistically significant at *p* ≤ 0.05 unless otherwise stated. To evaluate the diagnostic performance of the three biomarkers CD14, E-FABP, and CCL11 we constructed receiver-operating-characteristic (ROC) curves comparing patients with IgE CMA to non-IgE CMA. ROC analyses were performed in R (version 4.4.0) with the ROCR package. For each biomarker we calculated the area under the ROC curve (AUC) with 95% confidence interval (CI) and tested the null hypothesis of AUC = 0.50.
