# Supplementary material for: Skin and gut microbiota composition and immune regulatory response differentiate IgE and non-IgE cow’s milk allergy patients with atopic dermatitis
Source: iScience. 2025 Nov 4;28(12):113943. doi: 10.1016/j.isci.2025.113943 (PMC12719065; doi:10.1016/j.isci.2025.113943)
Supplement: Document S1. Figures S1–S11 and Tables S1–S4 [file mmc1.pdf]

iScience, Volume *28*

Supplemental information

Skin and gut microbiota composition and immune regulatory response differentiate IgE and non-IgE cow's milk allergy patients with atopic dermatitis

Tomas Thon, Eliska Kopelentova, Dagmar Srutkova, Stepan Coufal, Jakub Kreisinger, Filip Rob, Zuzana Reiss, Miloslav Kverka, Stepanka Capkova, Jana Cadova, Lucie Bulantova, Vojtech Bystry, Anna Sediva, Helena Tlaskalova-Hogenova, Zuzana Jiraskova Zakostelska, and Andrea Polouckova

Supplementary Material

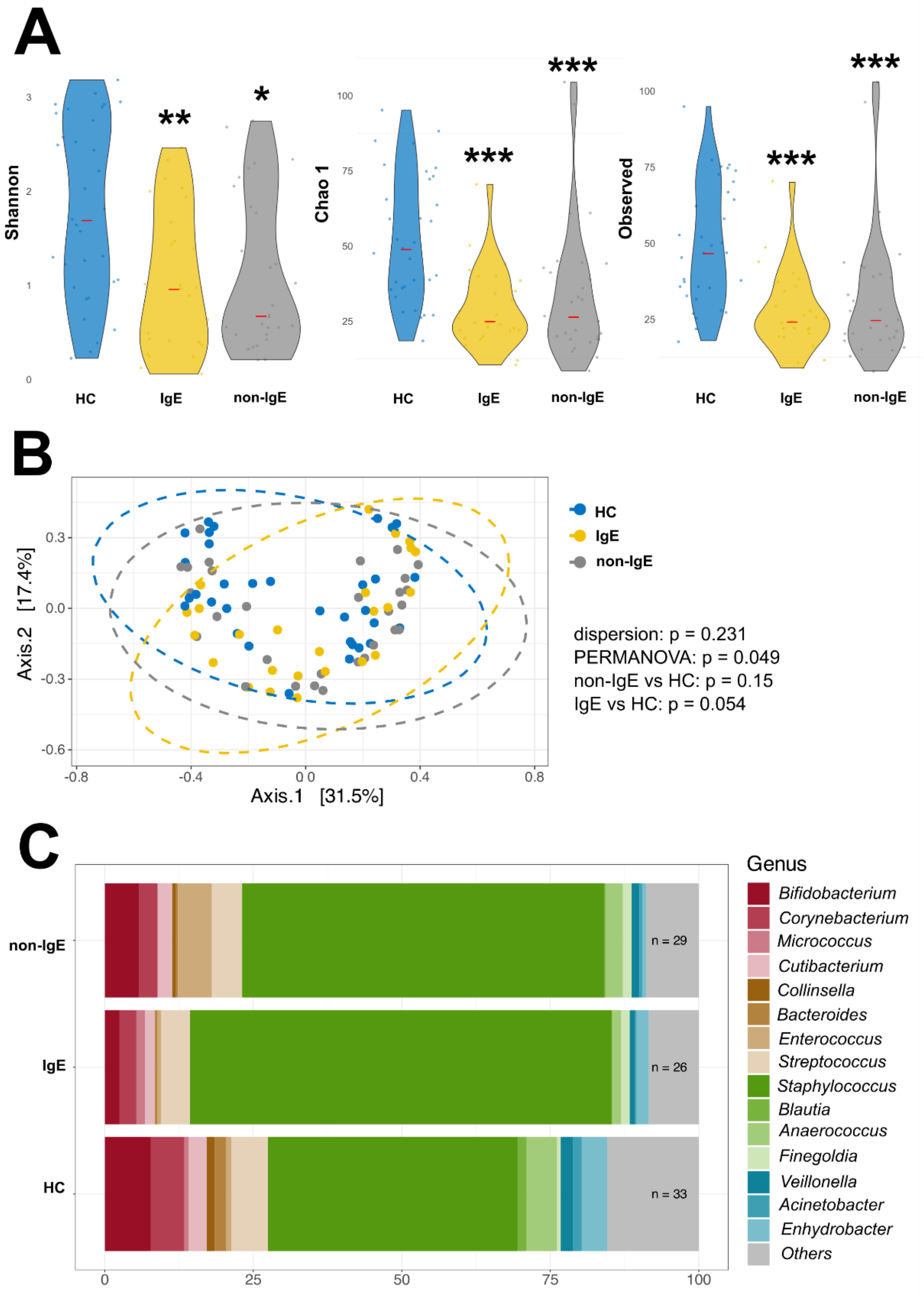

Figure S1: Bacteria in the popliteal fossa of patients with non-IgE CMA differ from patients with IgE CMA and HC. Related to Figure S3. *(**A**) Alpha diversity expressed as Shannon diversity index, Chao1, Observed taxa. (**B**) Beta diversity was calculated using Bray-Curtis dissimilarities and visualized by Principal Coordinate Analysis (PCoA) where each point represents one patient or healthy control and tested with PERMANOVA. (**C**) Abundances of the 15 most abundant bacterial genera. Statistically significant differences between groups are marked with ∗. These data display results without covariates for age and sex. (∗p < 0.05, ∗∗p < 0.01, ∗∗∗p < 0.001)*

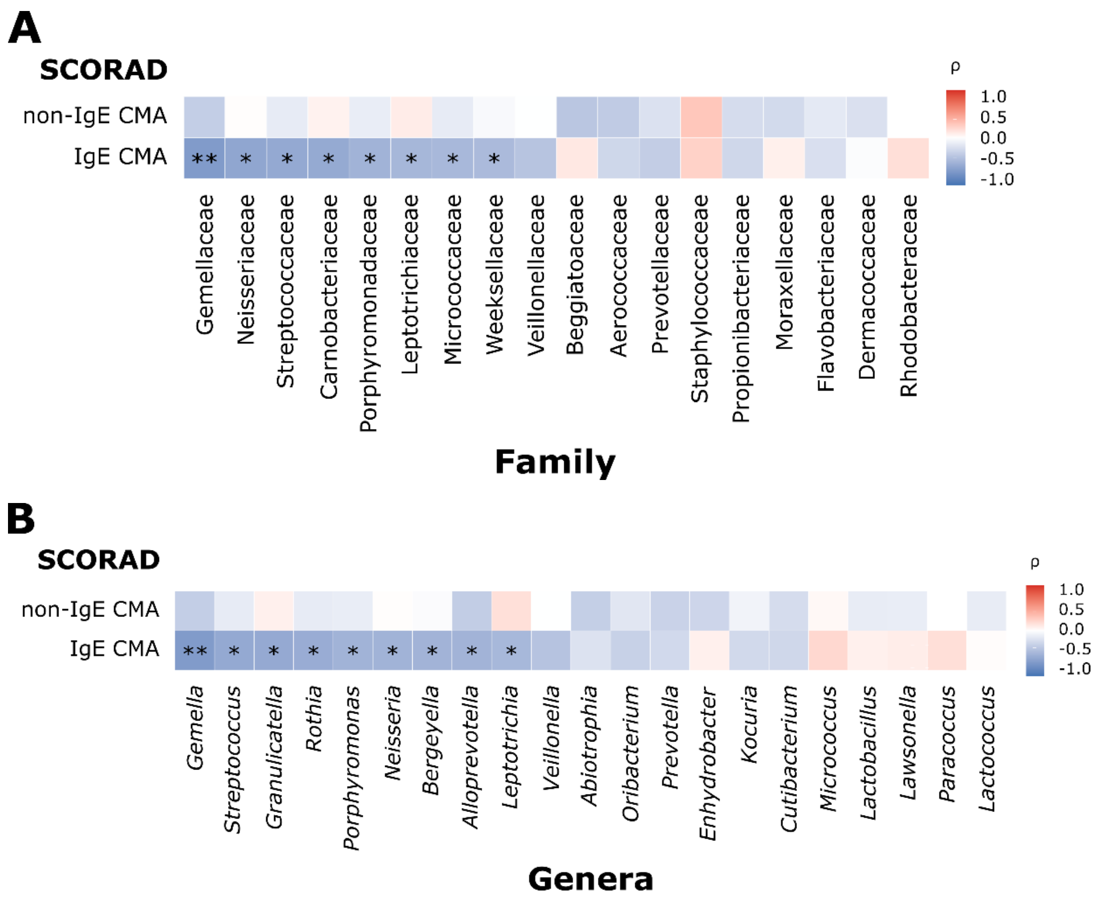

Figure S2. Correlation patterns between the severity of atopic dermatitis described by SCORAD and cheek bacterial abundance in IgE and non-IgE CMA patients. Related to Table 4. *For each bacterial (A) family or (B) genus identified, we calculated Pearson partial correlation coefficients with SCORAD scores, controlling for potential confounding variables (age and sex as covariates) using the ppcor function in R. P-values were adjusted for multiple testing. Positive correlations are shown in red, negative correlations in blue. Statistically significant differences are marked with ∗ (∗p < 0.05, ∗∗p < 0.01).*

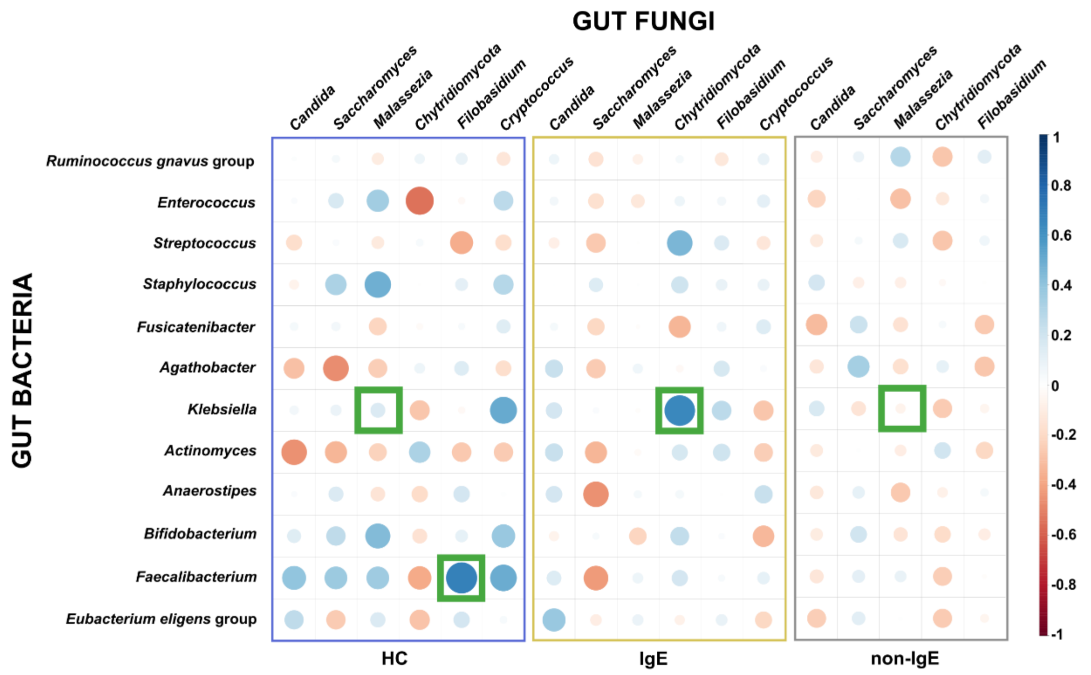

Figure S3. Correlation patterns between gut bacteria and fungi in patients with IgE or non-IgE CMA and HC. Related to Figures 1 and 2. *Pearson correlation for the relative abundances of bacterial and fungal populations of the patients with IgE or non-IgE CMA or HC. The correlations were calculated separately for each sample group – HC (left), patients with IgE CMA (center), and patients with non-IgE CMA (right). Positive correlations are represented by blue dots; negative correlations are represented by red dots. Significant differences are marked with green squares, while non-significant correlations ($p > 0.05$) are blank.*

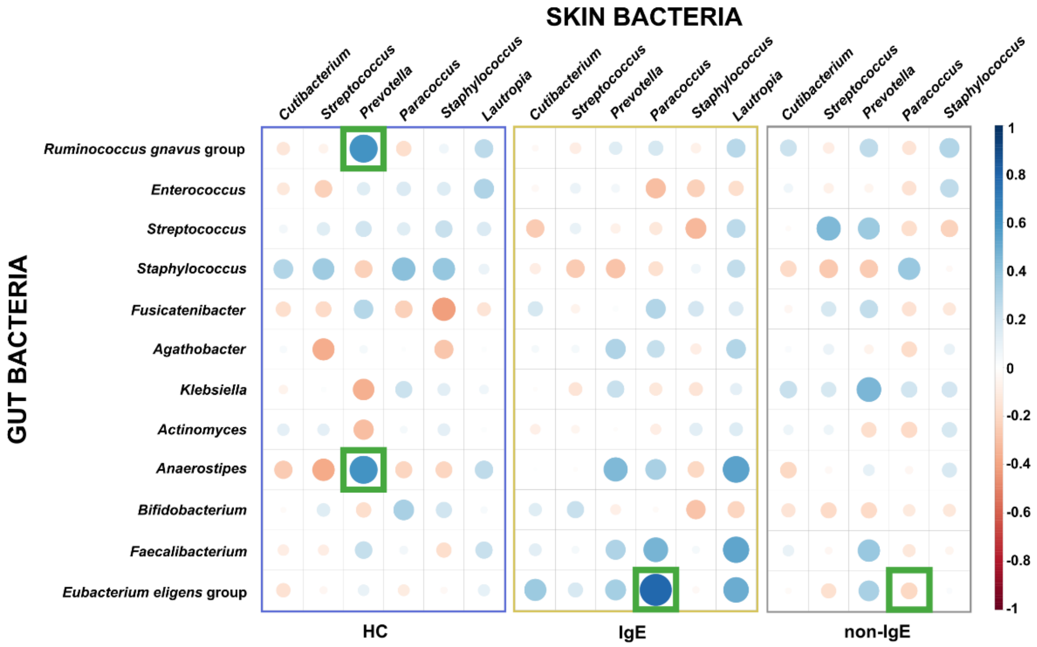

Figure S4. Gut-skin bacteria correlation pattern in relation to patients with IgE or non-IgE CMA or HC. Related to tables 1 and 3. *Pearson correlation for the relative abundances of bacterial populations in the gut and skin of the patients with IgE or non-IgE CMA or HC. The correlations were calculated separately for each sample group – HC (left), patients with IgE CMA (center), and patients with non-IgE CMA (right). Positive correlations are represented by blue dots; negative correlations are represented by red dots. Significant differences are marked with green squares, while non-significant correlations (p > 0.05) are blank*

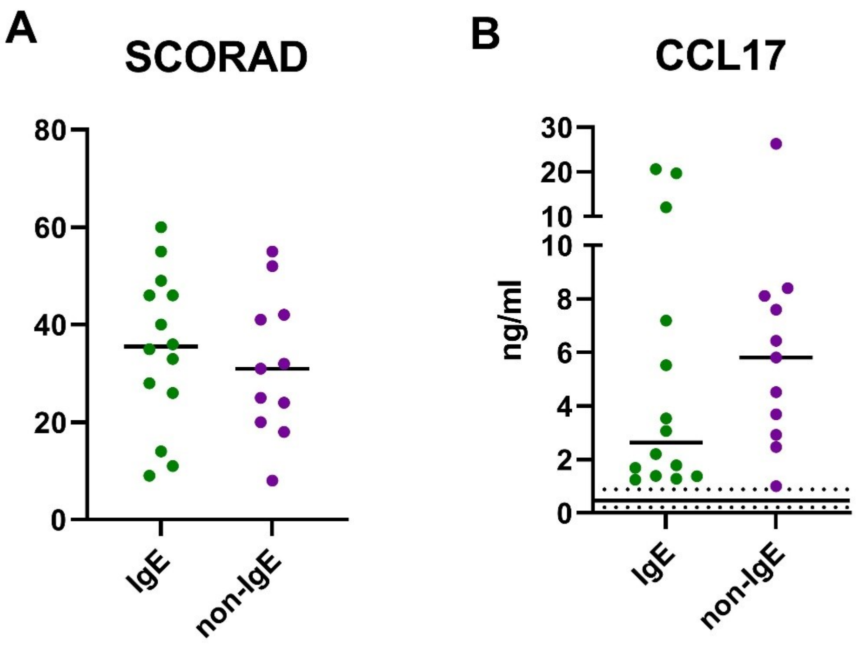

Figure S5*. *Related to Figure 4. *(A) Comparison of SCORAD values between IgE and non-IgE CMA patients in whom we characterized PBMC and serum biomarkers. (**B**) Changes in CCL17 serum levels between IgE and non-IgE CMA patients. The statistical differences between the two groups were analyzed using the non-parametric Mann-Whitney test. Black lines depict the median (full line) and 95% CI (dotted line) of healthy controls (HCs). IgE CMA patients (n=14), non-IgE CMA patients (n=11), HC (n=5-7).*

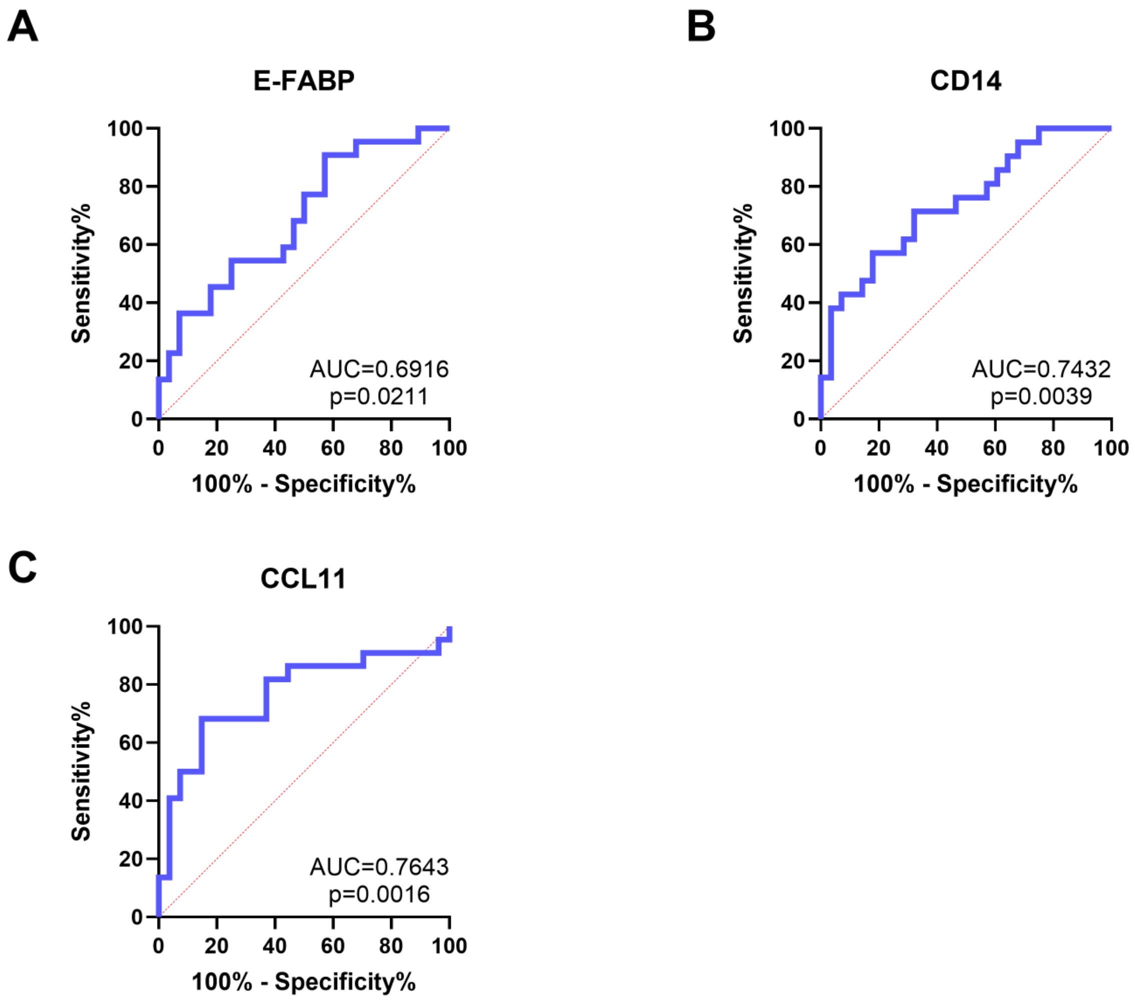

Figure S6. Composite ROC curve analysis discriminating IgE from non-IgE CMA patients. Related to Figure 4. *Composite ROC curve analysis for (**A**) E-FABP, (**B**) CD14, (**C**) CCL11.*

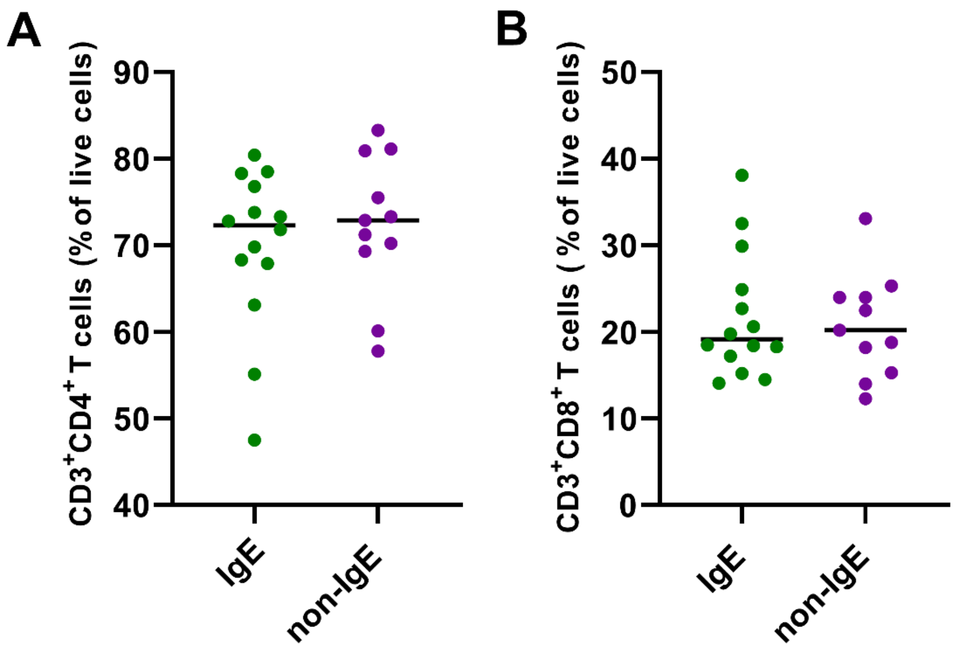

Figure S7. *Flow cytometry analysis of T cell staining for CD3^{+}CD4^{+}* ***(A)****, CD3^{+}CD8^{+}* ***(B)*** *in patients with IgE and non-IgE CMA in whom we characterized PBMC and serum biomarkers. The statistical differences between two groups were analyzed using the non-parametric Mann-Whitney test.*

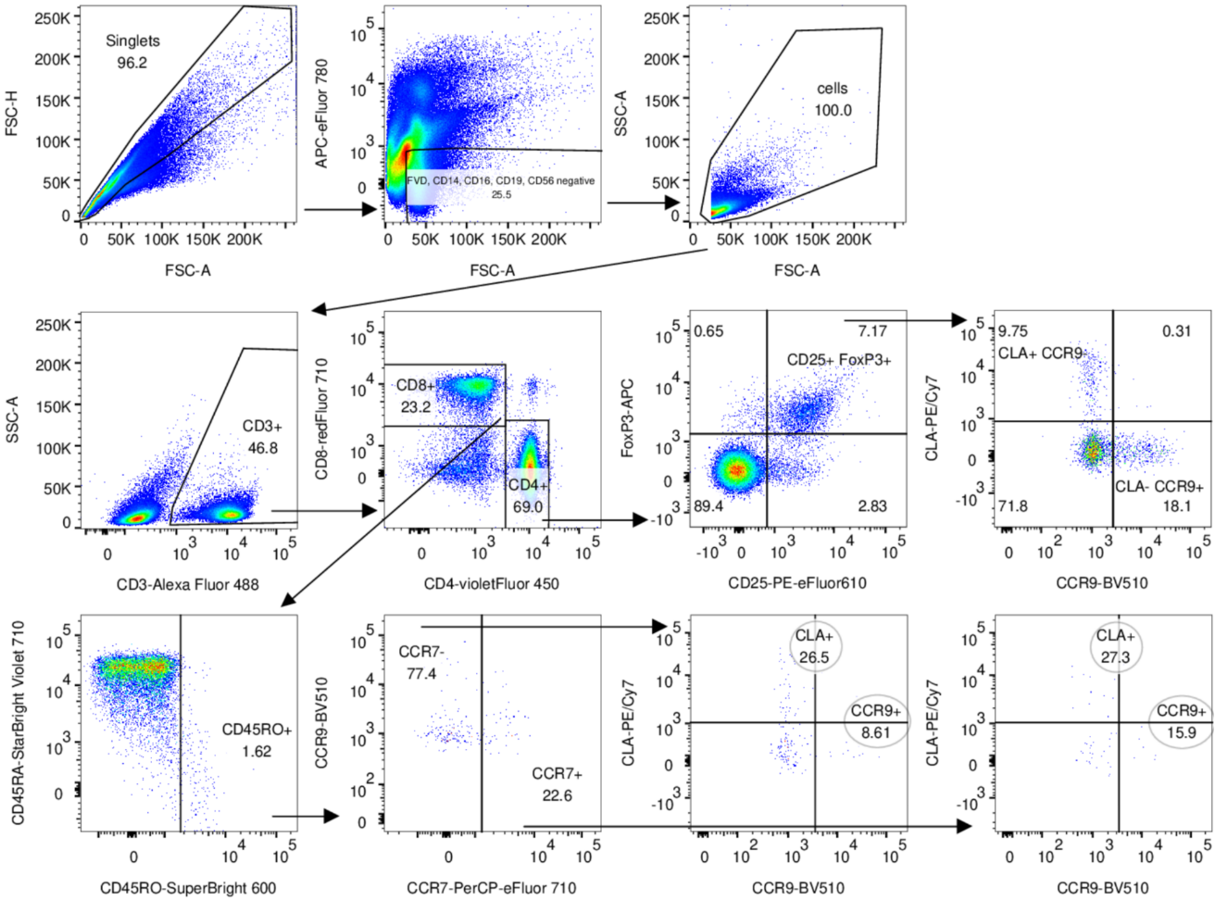

Figure S8. Gating strategy for T cells describing PBMC of children with IgE and non-IgE CMA and AD. Related to Figures 5 and 6.

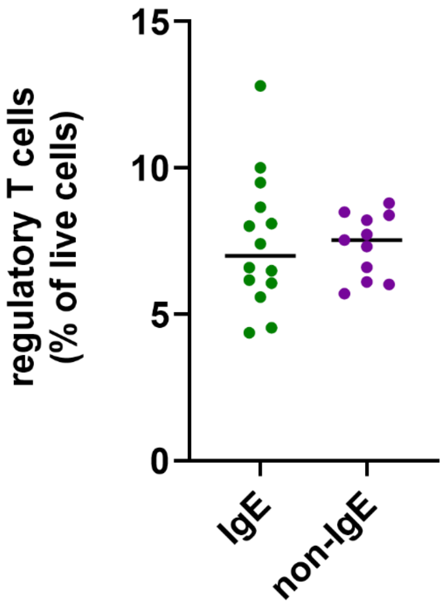

Figure S9. *Flow cytometry analysis of Tregs CD3$^+$CD4$^+$CD25$^+$FoxP3$^+$ in patients with IgE and non-IgE CMA. The statistical differences between two groups were analyzed using the non-parametric Mann-Whitney test.*

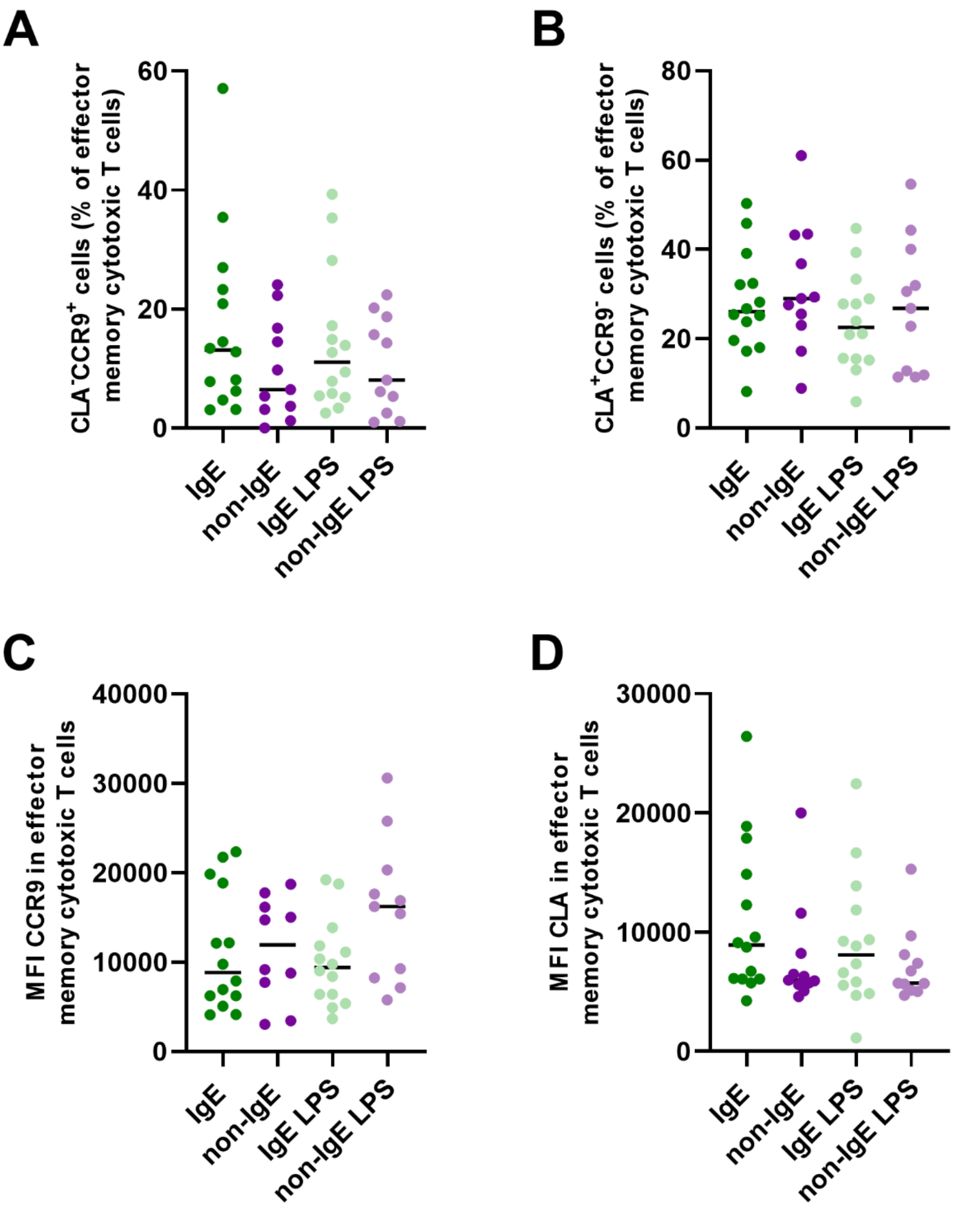

***Figure S10**. **There are no changes in homing to gut or skin on central effector T cytotoxic lymphocytes upon stimulation by LPS**. Phenotypic profile of circulating $CD8^+CD45RO^+CCR7^-$ T lymphocytes homing to gut ($CCR9^+$) (**A**) or to the skin (CLA^+) (**B**) in LPS stimulated and unstimulated cells. Expression of surface markers of $CD8^+CD45RO^+CCR7^-$ T lymphocytes homing to gut ($CCR9^+$) (**C**) or to the skin (CLA^+) (**D**) in LPS stimulated and unstimulated cells. Mean Fluorescence Index (MFI) for each of the indicated markers ($CCR9^+$ or CLA^+) was evaluated. The statistical differences between the two groups were analyzed using the non-parametric Mann-Whitney test. Statistically significant differences between groups or between time points are marked with * (*$p < 0.05$). IgE CMA (n=14); non-IgE CMA (n=11); LPS - PBMC stimulated with LPS*

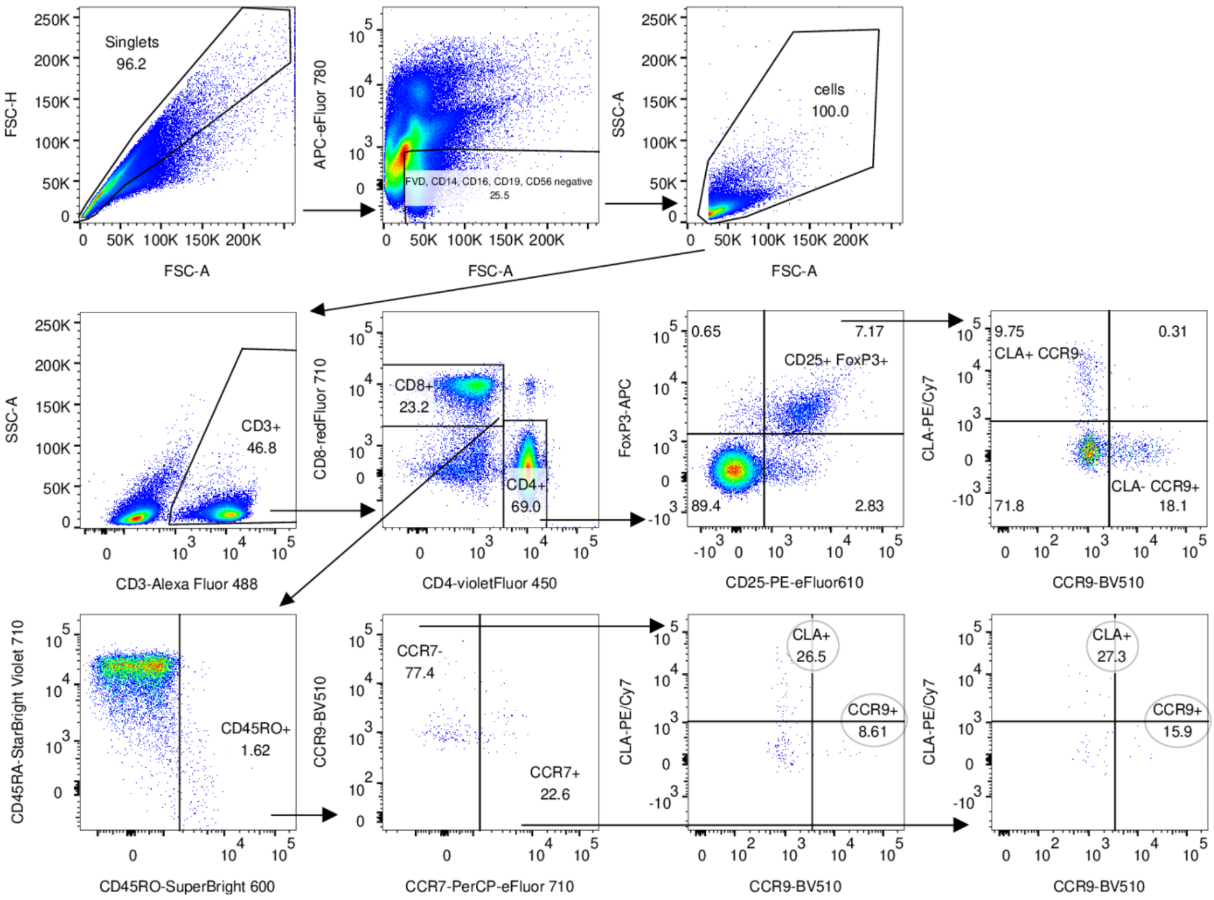

Figure S11. Gating strategy for dendritic cells describing PBMC of children with IgE and non-IgE CMA and AD. Related to Figure 7.

**Skin – bacteria (popliteal fossa)**		
**Family**	**IgE vs. HC (adjusted p values)**	**non-IgE vs. HC (adjusted p values)**
Bacteroidaceae	0.0039 ↓	0.1434
Bifidobacteriaceae	0.0379 ↓	0.1559
c,Alphaproteobacteria_o,Unassigned	0.0069 ↑	0.0704 ↑
Dermacoccaceae	0.0069 ↓	0.5950
Enterobacteriaceae	0.0151 ↓	0.0315 ↓
Family XI	0.0251 ↓	0.3744
Gemellaceae	0.0075 ↓	0.8500
Lactobacillaceae	0.0355 ↓	0.8539
Lachnospiraceae	0.0039 ↓	0.1434
Moraxellaceae	0.0039 ↓	0.0704 ↓
p,Proteobacteria_c,Unassigned	0.0069 ↑	0.0018 ↑
Propionibacteriaceae	0.0183 ↓	0.5735
Pseudomonadaceae	0.0069 ↓	0.5950
Rhodobacteraceae	0.0065 ↓	0.0704 ↓
Weeksellaceae	0.0075 ↓	0.4303
**Genus**	**IgE vs. HC (adjusted p values)**	**non-IgE vs. HC (adjusted p values)**
[Ruminococcus] gnavus group	0.0021 ↓	0.0930
Acinetobacter	0.0267 ↓	0.4915
Anaerococcus	0.0128 ↓	0.1440
Bacteroides	0.0051 ↓	0.2036
Bergeyella	0.0406 ↓	0.7367
Blautia	0.0161 ↓	0.6208
c,Alphaproteobacteria_o,Unassigned	0.0161 ↑	0.1440
Cutibacterium	0.0250 ↓	0.6208
Dermacoccus	0.0140 ↓	0.6208
Enhydrobacter	0.0140 ↓	0.1440
Escherichia-Shigella	0.0059 ↓	0.0930
Gemella	0.0140 ↓	0.8319
Lactococcus	0.0035 ↓	0.6541
p,Proteobacteria_c,Unassigned	0.0161 ↑	0.0076 ↑
Paracoccus	0.0099 ↓	0.0630 ↓
Pseudomonas	0.0109 ↓	0.6208

Table S1. Composition of the skin microbiota of popliteal fossa is altered in infants with IgE and non-IgE CMA compared to HC. Related to Table 4. *↑↓ - indicates whether a particular bacterial family or genus is increased or decreased in both the IgE CMA and non-IgE CMA groups compared to a HC group. Patients with IgE CMA (n=26); patients with non-IgE CMA (n=29); HC (n=33). The P-values in this analysis are adjusted for gender and age in months. Statistically significant differences between groups are considered when p < 0.05.*

	Patients with IgE CAM (n=14)	Patients with non-IgE CAM (n=12)	Healthy controls (n=7)
**Median of age in months (1.q; 3.q)**	6.8 (4.7; 8.9)	4.7 (3.3; 6.7)	8.3 (7.4; 11.8)
**Sex (%)**			
**female**	8 (57.1)	5 (41.7)	2 (28.6)
**male**	6 (42.9)	7 (58.3)	5 (71.4)
**SCORAD (%)**			-
**mild**	3 (21.4)	5 (41.7)	
**moderate**	9 (64.3)	5 (41.7)	-
**severe**	2 (14.3)	2 (16.7)	-
**Other type of food allergy (%)**			-
**yes**	14 (100.0)	10 (83.3)	
**no**	0 (0.0)	2 (16.7)	-
**Type of birth (%)**			
**vaginal**	11 (78.6)	9 (75.0)	6 (85.7)
**caesarean**	3 (21.4)	3 (25.0)	1 (14.3)
**Breastfeeding (%)**			
**yes**	13 (92.9)	11 (91.7)	5 (71.4)
**no**	1 (7.1)	1 (8.3)	2 (28.6)
**Mothers' history of allergic diseases (%)**			
**yes**	7 (50.0)	9 (75.0)	2 (28.6)
**no**	7 (50.0)	2 (16.7)	5 (71.4)
**NA**	0 (0.0)	1 (8.3)	0 (0.0)
**Fathers' history of allergic diseases (%)**			
**yes**	10 (71.4)	7 (58.3)	0 (0.0)
**no**	4 (28.6)	4 (33.3)	7 (100)
**NA**	0 (0.0)	1 (8.3)	0 (0.0)
**Form of AD (%)**			
**extrinsic**	12 (85.7)	8 (66.7)	-
**intrinsic**	2 (14.3)	4 (33.3)	-
**Onset of tolerance to milk in 1 year (%)**			
**yes**	0 (0.0)	1 (8.3)	-
**no**	14 (100)	11 (91.7)	-
**Gastrointestinal manifestations (%)**			
**yes**	5 (35.7)	3 (25.0)	-
**no**	9 (64.3)	9 (75.0)	-
**Stridor (%)**			
**yes**	4 (28.6)	2 (16.7)	-
**no**	10 (71.4)	10 (83.3)	-
**Allergic rhinitis (%)**			
**yes**	8 (57.1)	5 (41.7)	-
**no**	6 (42.9)	7 (58.3)	-
**Inhalant allergens (%)**			
**yes**	11 (78.6)	6 (50.0)	-
**no**	3 (21.4)	5 (41.7)	-
**NA**	0 (0.0)	1 (8.3)	-
**Hypogammaglobulinemia (%)**			
**yes**	10 (71.4)	11 (91.7)	-
**no**	4 (28.6)	1 (8.3)	-
**SCIg therapy (%)**			
**yes**	4 (28.6)	2 (16.7)	-
**no**	10 (71.4)	10 (83.3)	-

Table S2. Summary of anthropometric and clinical parameters of patients with non-IgE CMA and patients with IgE CMA in whom we analyzed serum biomarkers and characterized PBMC. Related to Figures 4, 5, 6, and 7.

Table S3. Fluorochrome-labeled antibodies used for T cell panel. Related to Figures 5 and 6.

**Antibody**	**Clone**	**Fluorochrome**	**Manufacturer**	**Cat. No**
FVD	-	eFluor™ 780	Thermo Fisher Scientific, Invitrogen, eBioscience™	65-0865-14
CD3	OKT3	Alexa Fluor™ 488	Thermo Fisher Scientific, Invitrogen, eBioscience™	53-0037-42
CD4	RPA-T4	violetFluor™ 450	Cell Signaling Technology	26755S
CD8	RPA-T8	redFluor™ 710	Abcam	ab241937
CD25	CD25-4E3	PE-eFluor™ 610	Thermo Fisher Scientific, Invitrogen, eBioscience™	61-0257-42
FoxP3	236A/E7	Allophycocyanin (APC)	Thermo Fisher Scientific, Invitrogen, eBioscience™	17-4777-42
CCR7	3D12	PerCP-eFluor™ 710	Thermo Fisher Scientific, Invitrogen, eBioscience™	46-1979-42
CD45RA	F8-11-13	StarBright Violet 710	Bio Rad	MCA88SBV710
CD45RO	UCHL1	Super Bright™ 600	Thermo Fisher Scientific, Invitrogen, eBioscience™	63-0457-42
CCR9	C9Mab-1	Brilliant Violet 510	BD Biosciences	752588
CLA	HECA-452	PE/Cyanine7	BioLegend	321316
CD14	61D3	APC-eFluor™ 780	Thermo Fisher Scientific, Invitrogen, eBioscience™	47-0149-41
CD16	3G8	APC-eFluor™ 780	Thermo Fisher Scientific, Invitrogen, eBioscience™	47-0166-42
CD19	HIB19	APC-eFluor™ 780	Thermo Fisher Scientific, Invitrogen, eBioscience™	47-0199-42
CD56	CMSSB	APC-eFluor™ 780	Thermo Fisher Scientific, Invitrogen, eBioscience™	47-0567-42

Table S4. Fluorochrome-labeled antibodies used for dendritic cell panel. Related to figure 7.

Antibody	Clone	Fluorochrome	Manufacturer	Cat. No
FVD	-	eFluor™ 780	Thermo Fisher Scientific, Invitrogen, eBioscience™	65-0865-14
CD11c	3.9	Fluorescein isothiocyanate (FITC)	Thermo Fisher Scientific, Invitrogen, eBioscience™	11-0116-42
HLA-DR	LN3	eFluor™ 506	Thermo Fisher Scientific, Invitrogen, eBioscience™	69-9956-41
CD123	6H6	Phycoerythrin (PE)	Thermo Fisher Scientific, Invitrogen, eBioscience™	12-1239-42
CD11b	ICRF44	Alexa Fluor® 700	BioLegend	301356
CD209	9E9A8	Brilliant Violet 421™	BioLegend	330118
CLEC7A	15E2	PerCP-eFluor™ 710	Thermo Fisher Scientific, Invitrogen, eBioscience™	46-9856-42
CD3	UCHT1	APC-eFluor™ 780	Thermo Fisher Scientific, Invitrogen, eBioscience™	47-0038-42
CD56	CMSSB	APC-eFluor™ 780	Thermo Fisher Scientific, Invitrogen, eBioscience™	47-0567-42
CD16	3G8	APC-eFluor™ 780	Thermo Fisher Scientific, Invitrogen, eBioscience™	47-0166-42
CD19	HIB19	APC-eFluor™ 780	Thermo Fisher Scientific, Invitrogen, eBioscience™	47-0199-42
CD14	M5E2	Brilliant Violet 605™	BioLegend	301834

List of abbreviations

E-FABP epidermal fatty acid binding protein, I-FABP intestinal fatty acid binding protein, L-FABP liver fatty acid binding protein, CD14 cluster of differentiation 14, ASCA IgG anti-Saccharomyces cerevisiae immunoglobulin G antibodies, ASCA IgA anti-Saccharomyces cerevisiae immunoglobulin A antibodies, IgE immunoglobulin E, CCL11 CC chemokine motif ligand 11, IL-18 interleukin 18.
